# Tyrosine Kinase Receptors in Oncology

**DOI:** 10.3390/ijms21228529

**Published:** 2020-11-12

**Authors:** Jorge Esteban-Villarrubia, Juan José Soto-Castillo, Javier Pozas, María San Román-Gil, Inmaculada Orejana-Martín, Javier Torres-Jiménez, Alfredo Carrato, Teresa Alonso-Gordoa, Javier Molina-Cerrillo

**Affiliations:** 1 Medical Oncology Department, University Hospital Ramon y Cajal, 28034 Madrid, Spain; elementjorge00@gmail.com (J.E.-V.); jj27sc@gmail.com (J.J.S.-C.); pozas.javier@gmail.com (J.P.); mariasanro@gmail.com (M.S.R.-G.); inma.orejana@gmail.com (I.O.-M.); javier.torres.jim@gmail.com (J.T.-J.); 2 Medical Oncology Department, Ramón y Cajal Health Research Institute (IRYCIS), CIBERONC, Alcalá University, University Hospital Ramon y Cajal, 28034 Madrid, Spain; acarrato@telefonica.net (A.C.); javier.molinace@gmail.com (J.M.-C.)

**Keywords:** tyrosine kinase receptors, tyrosine kinase receptor inhibitors, tumorigenesis, research

## Abstract

Tyrosine kinase receptors (TKR) comprise more than 60 molecules that play an essential role in the molecular pathways, leading to cell survival and differentiation. Consequently, genetic alterations of TKRs may lead to tumorigenesis and, therefore, cancer development. The discovery and improvement of tyrosine kinase inhibitors (TKI) against TKRs have entailed an important step in the knowledge-expansion of tumor physiopathology as well as an improvement in the cancer treatment based on molecular alterations over many tumor types. The purpose of this review is to provide a comprehensive review of the different families of TKRs and their role in the expansion of tumor cells and how TKIs can stop these pathways to tumorigenesis, in combination or not with other therapies. The increasing growth of this landscape is driving us to strengthen the development of precision oncology with clinical trials based on molecular-based therapy over a histology-based one, with promising preliminary results.

## 1. Introduction

Since the discovery of tyrosine kinase receptors (TKR) in the 60′s, and their subsequent classification by families, it has been revealed how the function of these receptors is key in different aspects of the cell biology. In the meantime, it has been discovered how the alterations in the normal function of these receptors can play a key role in the development of tumors, from early stages of the disease to more advanced ones. The development of new drugs that are able to inhibit these receptors, and their downstream pathways, has been one of the main milestones in cancer treatment. A great variety of new TKR inhibitors (TKI) have been included in the therapeutic algorithm of different solid tumors after confirming their efficacy and improvement in patients ‘survival.

The aim of this review is to provide a comprehensive insight of the different roles of the TKRs within the cell biology, how their alteration can lead a tumorigenesis process and which drugs are currently available or under development to stop tumor progression.

## 2. Roles of TKRs in Physiologic Cell Biology 

There are nearly 60 different TKRs, which can be classified into 20 families according to their structure and their ligand. In general, in the absence of a ligand, the receptors are docked in the plasmatic membrane of the cell as monomers, which are the inactive form of the receptor. Ligand binding leads to the homo or heterodimerization of these receptors and their activation by a variety of mechanisms. Most of the receptors are activated by a process called transphosphorylation, while others are activated by conformational changes caused by the interaction of the tyrosine kinase domains. Both processes conclude in the phosphorylation of some tyrosine residues in the cytosolic side of the protein, which are the docking sites for some intracellular signalling proteins. These proteins bind to a specific site of the receptor by the reckoning of phosphotyrosine residues by a docking domain, which is usually the Src homologue 2 (SH2) or phosphotyrosine binding (PTB) type.

The following table details some of the TKR with their demonstrated functions [1,2,3,4,5,6,7,8,9,10,11,12,13,14,15,16,17] (Table 1).

Ras and Rho are two monomeric guanosine 5′ triphosphatases (GTPases) that are the most responsible for relaying signals from the membrane receptors. There are various types in the human cells, H, K and N being the most representative. These proteins are anchored to the cytosolic layer of the plasmatic membrane, where they send signals mainly to the nucleus [18].

Ras is able to activate a complex of proteins called mitogen-activated protein (MAP) kinases. This complex pathway is made of three proteins called Raf, mitogen-activated protein kinase kinase (MEK) and extracellular signal-regulated kinase (ERK). The three-component module begins with a MAP kinase called Raf. Ras recruits Raf to the plasmatic membrane and helps to activate it. Raf then activates MEK, which, in turn, activates ERK. To date, there are six distinct groups of MAPKs characterized in mammals; ERK1/2, ERK 3/4, ERK5, ERK7/8, Jun N-terminal (JNK)1/2/3 and the p38 isoforms α/β/γ (ERK6)/δ [19]. ERKs phosphorylate many cytoplasmic and nuclear kinases, phosphatases, transcription factors and cytoskeletal proteins. Sustained, but not transient activation of ERK signalling, promotes phosphorylation and stabilization of For, Jun, Myc, Egr-1 and cyclin D1 genes, thereby promoting cell-cycle entry. This stimulation also represses the expression of genes which inhibit proliferation; thus, controlling fundamental processes such as growth and proliferation [20,21,22].

The Rho family proteins are in charge of actin and microtubules of the cytoskeleton regulation. The human Rho GTPase family consists of 20 proteins divided into eight subfamilies, Rac, Rho, Cdc42 and RhoF/RhoD subfamilies being some of the most representative [23]. These proteins have a central role in cell shape, polarity, motility, endocytosis and chemotaxis as they depend on the actin cytoskeleton [24]. Rho proteins stimulate the activity of nucleation promoting factors (NPFs), such as WASP, which in turn activates actin-related proteins 2/3 (Arp2/3) and formin. These proteins are key in the regulation of stable new multimers of actin development [25]. Another important Rho effector is Rho-associated protein kinase (ROCK) which is involved in actin-myosin II filament assembly, cross-linking and generation of contractile forces by increasing the levels of phosphorylated myosin light chain (pMLC). Contraction promotes membrane blebbing, movement of the cell body and detachment of the cell rear [26]. Activation of Rho family proteins by TKR is key in the development of the nervous system, as it is involved in the migration of the neural cells and the development of the axons. The process of axon formation is guided by Eph and its TKRs, as the migration of neurons is guided by Trk family proteins [27].

Another kinase involved in recruiting proteins and activating signaling pathways is the phosphoinositide 3-kinase (PI 3-K). There are three classes of PI3K enzymes. Mammals express four class I catalytic isoforms (p110α, β, γ and δ). p110α and p110β are expressed ubiquitously, whereas expression of p110γ and p110δ is enriched in immune cells. Each one of these catalytic proteins forms a dimer with a regulatory subunit that modulates the activity and sub-cellular localization of the complex. PI-3K can be activated by Tyrosine kinase receptors (RTKs) by direct interaction with the receptor, as three of the class I isoforms, (p110α, p110β, p110δ, known as the class Ia subgroup) associate with regulatory subunits whose SH2 domains bind to phosphotyrosine residues present in TKRs. Each isoform has a domain that can interact with members of the Ras or Rho family. p110α, p110 γ and p110δ bind to Ras family members whereas p110β interacts with the Rac/Cdc42 subfamily [28]. PI-3Kinases catalyze the formation of phosphoinositide (being PI(3,4,5)P3 the most important), which constitute a substrate of a wide range of proteins with PH domains, which in turn act as second messengers of the signal. Those family proteins are, among others, the Ser/Thr protein kinases, Btk/Itk/Tec subfamily, Insulin Receptor Substrate 1 (IRS-1), regulators of small G-proteins, some cytoskeletal proteins or the mammalian phosphatidylinositol-specific phospholipase C (PI-PLC) [28]

A relevant member of this family is Protein kinase B (PKB or Akt), which plays a key role in cell survival and growth promotion in many cell types. Akt is regulated by PDK1 and mTOR complex 2. mTOR phosphorylates Akt on serine 473, so it changes its conformation, and then is phosphorylated by PDK1 on threonine 308. This process leads to the dissociation of Akt from the plasma membrane and now is able to activate many other downstream effectors. There are over 100 reported in the literature. These substrates are involved in cell proliferation, metabolism, survival and motility. Glycogen synthase kinase 3 (GSK3) was the first substrate reported of Akt. GSK3 is generally active in the absence of exogenous signals, and the substrates of GSK3 are usually down-regulated or degraded under GSK3-mediated phosphorylation. Thus, growth factor signalling trough Akt positively regulates these targets, such as the B cell Lymphoma (BCL-2) family member Induced myeloid leukemia cell differentiation protein (MCL-1), c-myc and Bad. Bad is an anti-apoptotic protein. This way, the activation of PI 3-K leads to the inhibition of the apoptosis, in turn, activating cell survival [28,29,30]. GSK3 also regulates cellular metabolism, either by phosphorylation of metabolic enzymes (GS) or indirectly by the regulatory inhibition of transcription factors such as Sterol regulatory element-binding protein 1 (SREBP1c), Hypoxia-inducible factor 1-alpha (HIF1a), and Nuclear factor erythroid 2-related factor 2 (NRF2) [31,32]. Another substrate of Akt is the forkhead box (FOXO) family members, which are transcription factors. These transcription factors are usually localized in the nucleus and Akt signalling leads to translocation of these proteins out of the nucleus and thus inhibiting its transcriptional program. Akt, therefore, supresses transcription factors involved in the induction of apoptosis (Bim, PUMA, Fasl, TRADD, TRAIL, NOXA, BCL6), cell-cycle arrest (p21, p27, Cyclin G2, p15, p19), catabolism and growth inhibition (Sestrin3, MAP1LC), tissue-specific metabolic changes (PEPCK, G6PC), autophagy (LC3, BNIP3), stem cell maintenance and terminal differentiation (NeuroD), DNA repair (Gadd45), glucose and lipid metabolism (PPAR-γ), stress resistance (mnSOD), pluripotency (OCT4, SOX2), immune response (CCR7, FOXP3) and other cellular processes [31,33,34].

Another PI3-K substrate is mammalian Target of Rapamycin (mTOR), which represents a family of serine/threonine kinases that act through two distinct protein complexes, named mTORC1 and mTORC2. These two proteins share some common subunits, such as the mTOR kinase, mLST8, dishevelled, EGL-10, DEPTOR, Tel2 and Tti1. They are different in terms of rapamycin sensitivity (mTORC1 is sensitive in contrast to mTORC2), binding components, sub-cellular localization, regulation and downstream substrates [35]. mTOR1 contains the regulatory-associated protein of mTORC1 (RAPTOR), whereas mTORC2 contains the rapamycin-insensitive companion of mTOR (RICTOR) and mSIN1. RICTOR is essential for the function, substrate recognition and cellular location of mTORC2, while mSIN1 negatively regulates mTORC2 [36]. In terms of cellular localization, mTORC1 is localized in endosomal and lysosomal membranes while mTORC2 is localized in plasmatic and ribosomal membranes, where it interacts with other proteins as AGC, PKA, PKG and PKC and SGK family kinases [37]. Both of them are regulated by several stimuli such as growth factors, hormones, hypoxia, nutrients, but mTORC2 is more sensitive to extracellular growth factors whose molecular mechanism is not elucidated yet [35,38]. The mTORC1 pathway is regulated by signalling pathways discussed earlier, such as PI3K/Akt and Ras-MAPK. mTORC1 activation by Akt requires the activation of the following steps referenced before (mTORC2 activation, phosphorylation of Akt in Ser473 by mTORC2 and at Thr308 by PDK1) and the phosphorylation of TSC2 by activated Akt, which inhibits TSC1 and TSC2 combination. The activator of mTORC1, RHEB, is constitutively down-regulated by TSC1/2. The inhibition of TSC1/2 leads to the release of RHEB and mTORC1 activation in lysosomes [35,39]. TSC2 can also be phosphorylated by ERKs from the Ras-MAPK pathway, leading to RHEB releasing and mTORC1 activation [40]. Activated mTORC1 will activate another downstream effector, such as 4EBP1, S6K1 and SREBP. This allows the eucaryotic initiation factors 3 and 4 (eIF-4E and eIF-3) release in order to intensify ribosome genesis [35,41]. mTORC1 also regulates several proteins, such as HIF1α, PP2A, glycogen synthase and STAT3. The promotion of protein, lipid and nucleotide synthesis is important for the G1-S phase transition and cell-size [35,42]. In contrast, two effectors of mTORC2 are SGK and protein kinase C (PKC). Activation of PKC will lead to changes in cytoskeleton, thus regulating cell movements [35,38].

**Figure 1:**
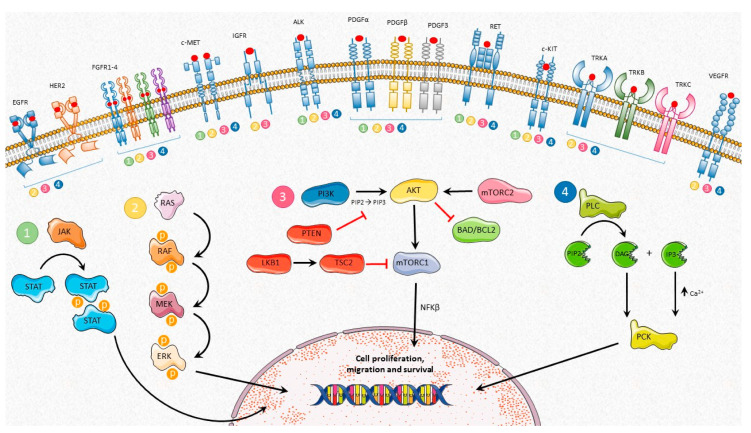
Above are represented the interaction between various signaling pathways activated through tyrosine kinase receptors (TKR) and involved in tumor proliferation. (1) Once a ligand binds to the receptor, two STAT proteins are phosphorylated by JAK forming a dimer which enters the nucleus, causing the transcription of target genes. (2) After the TKR is activated by a ligand, Ras dimerizes and binds Raf, promoting Raf activation. Active Raf phosphorylates and activates MEK1/2 which induces ERK1/2 activation, leading to transcription activation. (3) PI3K phosphorylates phosphatidyl inositol-bisphosphate (PIP2) to PIP3, a process that can be reversed by the action of PTEN. PIP3 causes the activation of Akt in the plasma membrane, thereby activating the mTOR complex, one of the major pathways involved in tumorigenesis. (4) PLC hydrolyzes PIP2, in this way forming diacylglycerol (DAG) and PIP3, which activate PKC and intracellular calcium mobilization, respectively.

Another relevant pathway activated by TKR is the PKC/phospholipase C-γ. The phospholipase acts on a phosphorylated inositol phospholipid (a phosphoinositide) called phosphatidylinositol 4,5-bisphosphate [PI(4,5)P2], which is present in the inner half of the lipid bilayer. The activated phospholipase then cleaves the PI(4,5)P2 to generate two products: inositol 1,4,5-trisphosphate (IP3) and diacylglycerol. At this step, the signaling pathway splits into two branches. IP3 leaves the plasma membrane and diffuses into the endoplasmic reticulum and binds to specific Ca^2+^ channels. This leads to the release of the Ca^2+^ stored in the reticulum, and the increase of cytosolic Ca^2+^ concentrations+. While this ion’s concentrations are raising, the other product, diacylglycerol, can act as a second messenger, activating the PKC, so-called because it is Ca^2+^ dependent. The initial rise of cytosolic Ca^2+^ leads to its translocation to the plasma membrane to be activated by Ca^2+^ and diacylglycerol [1,43]. There are at least 10 different isoforms of PKC, some are ubiquitously expressed whereas others are restricted to certain tissues. PKC is essential to smooth muscle contraction and proliferation [43]. Figure 1 summarizes which signaling pathways are activated by each receptor.

It is necessary for the cell to regulate the diversity of signals transduced by its receptors. Cells are desensitized to an extracellular signal by receptor sequestration, receptor down-regulation, receptor inactivation, inactivation of the signal protein and by the production of an inhibitory protein. We will review how these processes regulate downstream signalling of the TKRs. Once activated in the plasma membrane, TKRs are usually endocytosed. This endocytosis is regulated by extracellular ligand concentration, as seen in EGFR. Higher ligand concentrations lead to receptor endocytosis by non-clathrin mediated endocytosis (NCE) while lower concentrations lead to receptor endocytosis by clathrin-mediated endocytosis (CME) [44,45]. The molecular mechanisms underlying CME are defined with adaptor protein 2 (AP2) regulating EGFR clustering into clathrin-coated pits, initially required to optimize receptor phosphorylation and especially constrain EGFR signalling [46]. Once the receptor is internalized, EGFR is directed into early endosomes. At low ligand concentrations, the receptor does not undergo ubiquitination and is returned to the plasma membrane, so the receptor can be recycled. Through recycling, CME also serves to prolong and polarize EGRF signalling to specific cell regions, which are key to achieving a productive proliferative response [44,47]. At high EGFR concentrations, the receptor is ubiquitinated at the plasma membrane, thus leading to degradation at lysosomes. The endosomal sorting complexes required for transport (ESCRT) recognizes ubiquitination through a stepwise process leading to lysosomal endocytosis [48]. Higher EGFR concentrations also trigger ubiquitination and the internalization of EGFR directly into the endoplasmic reticulum in a non-clathrin-mediated process regulated by a protein called Reticulon-3, involved in the formation of contact sites between the plasma membrane and endoplasmic reticulum [30]. In the early endosomes, concentration of EGFR in the plasma membrane can also be unregulated, with the RNF11 transcription factor being translocated into the nucleus and inducing transcription of genes required for EGFR transport into the plasma membrane [44,49].

GTPases such as Ras and Rho are regulated by GTPase activating proteins (GAPs) and Guanine nucleotide exchange factors (GEFs). GEFs activate GTPases by promoting the release of bound GTP, which allows a new GTP to bind. Conversely increases the rate of GTP hydrolysis inactivating GTPases. ERK plays a key role in its negative regulation of the MAPK pathway. Activated ERK can inhibit Raf by direct phosphorylation and it is able to interfere with the coupling of Raf to a GEF called Sos, thus avoiding its activation [21,50,51]. Activated ERK can also promote the transcription of MKPs, which are able to dephosphorylate ERK activation sites, and the Sprouty family proteins, which interfere with Ras and Raf activation [21,52,53]. Rho is also controlled by Guanine nucleotide dissociation inhibitors (GDIs), which control Rho cycling between cytosol and membranes, holding them in an inactive form in the cytosol. Rho GTPases can also undergo phosphorylation by PKA, ROCK1, Src and Akt and, thus, be negatively regulated. Phosphorylation of RhoA by Erk2 facilitates ubiquitination of RhoA, leading to its degradation in the proteasome [23,54,55].

PTEN (phosphatase and tensin homologue: MMAC/TEP1) is a negative regulator of the PI-3K/Akt signalling. PTEN is a phosphoinositide phosphatase, acting as a direct antagonist of PI-3K, dephosphorylating PtdIns(3,4,5)P3 to form PtdIns(4,5)P2. Cellular localization of PTEN is crucial for its normal cellular function. In the cytoplasm, its main function is to regulate the cell cycle, apoptosis, cell adhesion and migration by antagonizing PI-3K/Akt/mTOR pathway. In the nucleus, PTEN plays a role in chromosomal stability and DNA double-strand break repair [56,57]. PTEN is regulated at a transcriptional level positively by EGR-1, PPAR-γ, ATF2 and p53, while is negatively regulated by CBF-1, NF-𝜅b, c-JUN and zinc-finger like proteins SNAIL and SLUG. At a post-transcriptional level, phosphorylation of PTEN by casein kinase 2 and GSK3β results in a decrease of phosphatase activity, but greater protein half-life [56,58]. mTORC1 is also an important regulator of the PI-3K/Akt signalling pathway. mTORC1 activation promotes Insulin receptor substrate 1 and 2 (ISR1 and ISR2) degradation through multiple phosphorylation on serine residues. ISR1 and ISR2 are scaffolding proteins that are able to link the insulin and IGF1 receptors to PI-3K activation. This way, PI-3K activation is dampened [31,59]. S6K, a substrate of mTORC1, once activated is able to phosphorylase RICTOR and Sin1, thus decreasing mTORC2-dependent phosphorylation of Akt T450 [31,60].

It is important to note that these major pathways and their regulators already discussed are cross-talking and redundant in cells. These cross-talking points are important to regulate downstream components activated by other pathways. MAPK pathway and PI-3K/Akt pathway interact at many points, as exemplified by Raf activation by Akt. Akt phosphorylases C-Raf and B-Raf, thus inhibiting ERK activation by Raf [61]. In addition, Erk activation can suppress RTK induction of the PI-3K pathway by phosphorylation of the GAB1 and GAB2 scaffolding proteins, in a similar way as we have discussed before [31,62]. Both pathways converge to regulate several downstream effectors and regulators as TSC2, FOXO, GSK3, RAPTOR and cellular processes [62]. However, both pathways can also act in a cooperative manner. For example, MAPK interacting kinases (MNK1 and MNK2) phosphorylate the cap-binding protein eIF4E to promote its ability to initiate transcription, and mTORC1-mediated phosphorylation of the 4E-BP proteins stimulates their release from the inhibitory binding of eIF4E and, thus, promoting transcription [62,63].

In summary, activation of TKRs gives rise to the activation of a series of complex signaling cascades, which are connected to regulate basic cellular processes such as replication, growth, differentiation, motility and communication with the cellular environment.

## 3. Roles of TKRs in Tumor Biology

As we have previously discussed, a main function of the TKR is to coordinate how the cells behave in their environment, regulating mitosis, differentiation or apoptosis. This harmonization between every member of an organism allows its survival and complexity. Alterations in RTKs can therefore bring about an evolutionary advantage of a given cell that will ultimately lead to a cell line with higher ability to survive in its environment. These evolutive advantages are summarized in the two basic properties of a cancer cell: The ability to divide independently and to invade other tissues [64].

In vitro assays have shown that cancer cells exhibit an altered phenotype with aberrations in shape, motility and in the way they integrate with their environment. It is characteristic of culture-grown cancer cells that their growth and duplication are not inhibited by contact with other cells and the absence of contact with the substrate, in contrast to normal cells. We can conclude that a characteristic of cancer cells is the alterations in extracellular signal processing. The essential mechanism underlying these aberrations are somatic and germline mutations of the DNA [1,64].

Every single gene is likely to have undergone mutation on about 10^10^ separate occasions in a human. Tumor cells accumulate mutations at a higher rate than normal cells, and that is why they are said to have genetic instability [65,66]. Thus, it is the progressive accumulation of mutations in key genes of cellular biology what gives rise to the development of a tumor. These genes are divided into proto-oncogenes and tumor-suppressor genes.

We will focus our attention on proto-oncogenes as some of these genes encode TKRs. A proto-oncogene is a normal gene that encodes a protein implicated in cellular growth, differentiation, or apoptosis, for example, the EGFR. There are plenty of mechanisms in which a proto-oncogene becomes an oncogene, being the most relevant the deletion or point mutation in the coding region or in the regulating region, the increase in the number of copies of the gene or chromosomic rearrangements [67]. One example is the amplification of ErbB2, which can form homo or heterodimers with other members of the ErbB family. The increase in the density of these receptors in the plasma membrane can lead to their dimerization in absence of a ligand and subsequent constitutive activation with sustained downstream signaling [68,69]. Other examples are the mutations in the extra or intracellular domains of the protein. Mutations in the extracellular domain of EGFR (i.e., EGFRvIII) can lead to a ligand-independent activation, but these mutations also cause an altered phosphorylation of the receptor at Cbl-binding sites, thus affecting receptor ubiquitination and turnover. This leads to increased signalling [70]. Mutations in the intracellular kinase domain also cause ligand-independent activation, leading to a kinase domain constitutively activated. For example, mutations in the activation loop of the EGFR receptor (L858R) promotes de-stabilization of the steady-state, thus converting to a more active state. These alterations also impair receptor ubiquitination affecting receptor trafficking [71,72]. These mutations that impair receptor intracellular trafficking and degradation/turnover can help to prolong the propagation of the signal and to locate receptors at specific membrane sites, stimulating cell motility and the formation of invadopodia [73,74]. Another example is the *EML4-ALK* fusion gene, resulting from the inversion of the short arm of the chromosome 2, gives rise to the expression of a chimeric protein with the tyrosine kinase domain constitutively activated [75]. Definitely, overactivation of the TKR represents a key pathogenic factor in the development of cancer.

The pathogenic role of TKR is not related only to activating or disrupting mutations but also to “non-canonical” activation of the receptors. Stressing stimuli, such as UV radiation, hypoxia or ionizing radiation. UV radiation leads to EGFR phosphorylation by p-38 MAPK at serine/threonine residues, which trigger receptor endocytosis and storage at endosomes. These receptors are not degraded and can be recycled again to the plasma membrane as p-38 MAPK is inactivated [44,76]. Hypoxia may lead to an upregulation of the EGFR gene transcription in the absence of genetic alterations [77]. Ionizing radiations increases EGFR expression and surprisingly, receptor endocytosis. However, as the receptor is endocytosed, is phosphorylated by PKC, impairing receptor ubiquitination and promoting its translocation to the nucleus.

Nuclear localization of the EGFR can be induced by EGF binding, Akt pathway, ionizing radiation and alkylating drugs as cisplatin [78]. Nuclear EGFR can bind to transcription factors as STAT3 to increase transcription several genes as iNOS, c-Myc and Cyclooxygenase-2 [78,79,80]. RNA helicase seems to be another nuclear-EGFR partner involved in EGFR-regulation of cyclin D1 [81]. EGFR can also play an important role in DNA repair, interacting with DNA-dependent protein kinase (DNA-PK) that leads to the repair of double-strand breaks of the DNA. Other described partners are p53 and MDC1 [82,83].

Furthermore, oncogenic signaling pathways can induce metabolic reprogramming in cancer cells supporting tumor growth. For example, EGFR has been involved in the regulation of several metabolic processes like biosynthesis of fatty acids, pyrimidines and glucose metabolism [84,85]. This is achieved indirectly by phosphorylating transcription factors like Myc, PI3K-Akt dependent nuclear translocation of sterol regulatory binding protein 1 (SREBP-1), phosphorylation of stearoyl-CoA desaturase-1, amongst others [86,87,88]. TKRs also collaborate in the metabolic drift to glycolysis as the main source of energy in cancer cells, known as the Warburg effect. GLUT-1, one of the main glucose transporters of the membrane, can be stabilized in the cell surface by the action of the PI3K/AKT/mTOR pathway, activated by EGFR mutant receptors [89]. EGFR also regulates the expression of Hexokinase 1 (HK1) and the activity of pyruvate kinase M2 (PKM2), two relevant enzymes in the glycolytic pathway [90]. The resulting increase in lactic acid, inhibits the activity of T-lymphocytes, supporting tumor immune escape [90].

The role of the TKRs is not only important in the tumor cell. Growing attention is given to the tumor microenvironment and its role in tumor progression. The tumor microenvironment is composed of stromal cells as fibroblasts, endothelial cells and adipocytes; immune cells such as lymphocytes, macrophages, monocytes and neutrophils; the extracellular matrix composed by macromolecules such as proteoglycans, structural proteins and glycoproteins; and other several components as growth factors, cytokines, chemokines and antibodies [91].

Angiogenesis is a hallmark of cancer, in response to a need of oxygen and nutrients from the bloodstream. Tumor vascularization requires cooperation between cancer cells, vascular endothelial cells, pericytes, BM-derived precursor cells, tumor-associated macrophages, mesenchymal stem cells (MSCs) and cancer-associated fibroblasts (CAFs). The main molecule responsible for angiogenesis is VEGF, although other important regulators are PDGF, FGF, placental growth factor and angiopoietin-1. Cancer cells are the main VEGF producers, although the other cell types described can also produce it [91,92,93]. VEGF transcription is stimulated under hypoxic conditions via HIF-transcription factors. VEGF binding to its receptor activates PI-3K/Akt/mTOR pathway at endothelial cells regulating its replication, differentiation and motility [37]. PDGF stimulated angiogenic process activates PI-3K/Akt/mTOR pathway, regulating the maturation of the newly formed vessels by attracting smooth muscle cells and pericytes [94].

CAFs are another important cellular population in tumors. These fibroblasts are different from normal fibroblasts and are present in aberrantly high numbers [92]. TGFβ, MCP1, PDGF, FGF have been implicated in CAF activation. Their origins are unclear, as there are studies suggesting an endothelial-to-mesenchymal transition origin [95] while others suggest its origin in epithelial-to-mesenchymal transition [96]. In tumors, their functions range from promoting tumor proliferation, angiogenesis, secretion of pro-inflammatory factors to tumor immunosuppression. CAFs are able to secrete VEGF and growth factors mentioned before [92].

Tumor-associated macrophages (TAMs) and myeloid-derived suppressor cells (MDSC) are important regulators of the tumorigenesis. Macrophages are attracted to the edge of the tumor by hypoxia-induced secretion of endothelin-2 and VEGF, as discussed before. Then, macrophage secretion of growth factors as CSF-1 and EGF can trigger the acquisition of an invasive phenotype by tumor cells [97]. TAMs also secrete VEFG, VEGF-C and VEGF-D. These last two correlate peritumoral inflammation and lymphangiogenesis [98]. TAMs usually show an M2 phenotype, playing an additional tumorigenic role by secretion of type II cytokines and, thus, promoting immune evasion [76,77]. MDSC can also promote tumor vascularization [99], but their key role is disrupting normal immunosurveillance by impairing antigen presentation by dendritic cells, T-cell activation, M1-macrophage polarization and inhibition of NK cytotoxicity [92].

In summary, the genetic mutations of the TKR give rise to aberrant proteins, constitutively active, or constitutively deregulated; which alters the transmission of signals in the cell, altering important cellular functions such as replication, migration and differentiation. This gives an evolutionary advantage with respect to the normal cells beyond the control of the usual regulatory mechanisms of cell proliferation. TKRs are also important in the regulation of the tumor microenvironment, where they play key pathogenic roles.

## 4. Tyrosine Kinase Receptor Inhibitors Developed for Cancer Treatment 

As of 22 July 2020, there are 61 tyrosine kinase inhibitors approved by the Food and Drug Administration (FDA) for the treatment of haematological and oncological malignancies with TKR disorders (http://www.brimr.org/PKI/PKIs.htm) (Table A1).

In the last two decades, a large number of drugs have been developed with the specific role of blocking intracellular signal pathways, that we could classify into two groups: monoclonal antibodies targeted against the self-receptor (extracellular portion) and small molecules with inhibitory activity against peptides harboring the tyrosine kinase function (intracellular signaling). In this section, we focus on drugs which directly inhibit the tyrosine kinase domain of the transmembrane receptor and have approval for the treatment of advanced solid tumors. A summarizing table of the different mutations of TKRs described below is supplied in Appendix A.

### 4.1. Epidermal Growth Factor Receptor (EGFR)

Targeting of EGFR family has been widely used as an effective therapeutic resource against cancer, since the discovery of an intimate involvement of this molecular pathway in cell proliferation, angiogenesis and motility. Specifically, the EGF receptor-1 (HER1 or ErbB1) and -2 (HER2 or ErbB2) blockade aims to inhibit the subsequent transduction signal and sequential steps: RAS/RAF/MEK, PI3K/Akt/mTOR, PKC, Src, and Jak/Stat. The majority of these transmembrane receptors behave as oncogenes when an activating mutation or overexpression occur, and become highly responsible of cell growth and survival “without limits” [100].

EGFR is involved in the oncogenic process of multiple malignancies. However, currently, we only have TKIs against this receptor for the treatment of a few cancers (especially, lung cancer). Only some EGFR activating mutations (exon 19 deletions, L858R point mutation in exon 21), and some resistance mutations are sensitive to therapeutic blockade with TKIs, so the identification of a driver mutation in EGFR before starting any systemic treatment is mandatory [100,101]. The two previous cited mutations account for 85% of all EGFR mutations, which lead to the kinase domain activation without the need for ligand binding [102]. Both mutations reduce the affinity of the kinase for adenosine triphosphate (ATP), allowing TKI to bind it.

The estimated incidence of EGFR mutation in advanced non-small cell lung cancer (NSCLC) is about 15–20% in the white race, and higher among the Asian female, and nonsmoker population [103].

The first EGFR TKIs were gefitinib and erlotinib (first generation, reversible binding). Subsequently, came afatinib and dacomitinib (second generation, irreversible binding). Gefitinib is a small molecule that selectively inhibits EGFR tyrosine kinase by binding to the ATP site, inhibiting receptor autophosphorylation and signal transduction. Three phase III trials (IPASS, West Japan Oncology Group 172 and North–East Japan Study Group 002) compared gefitinib with chemotherapy in advanced, EGFR-positive (activating mutations), Non Cell Samall Lung Cancer (NSCLC) patients, who were treatment-naïve. Gefitinib significantly improved the progression-free survival (PFS) and the objective response rates (ORR) in the three trials, although the difference in overall survival (OS) was not statistically significant [104,105,106,107]. Further analysis reported that EGFR overexpression did not seem to benefit from gefitinib. Combinations of gefitinib plus chemotherapy are not a standard in this setting. Gefitinib was approved by the FDA (2003) for the first-line treatment of patients with EGFR-positive NSCLC.

Erlotinib, with a similar mechanism of action to gefitinib, also demonstrated an increased PFS (versus platinum-based chemotherapy) in three trials: OPTIMAL (plus better ORR), EURTAC and ENSURE, but no statistically significant difference in OS was detected [108,109,110]. Erlotinib was approved by the FDA (2004) for the first-line treatment of EGFR exon 19 deletions or exon 21 substitution mutations in NSCLC. In 2010, the FDA extended the erlotinib indication as maintenance therapy for patients with advanced NSCLC whose disease had not progressed after four cycles of platinum-based chemotherapy in the first-line setting (EGFR mutation not previously identified). This decision was based on data from the phase III trial SATURN, which showed significantly better PFS with erlotinib maintenance versus placebo (45 weeks vs. 13 weeks, Hazard ratio [HR] 0.71) [111]. In 2016, the FDA modified the initial indication and limited the susceptible mutations to exon 19 deletions or exon 21 L858R substitution, because of the reported lack of survival benefit with erlotinib in the non-mutant population (results of the IUNO trial) [112]. In May 2020, erlotinib was also approved (in combination with ramucirumab) for first-line treatment of advanced NSCLC with EGFR exon 19 deletions or exon 21 (L585R) mutation. The RELAY trial compared the combination against erlotinib monotherapy, and reported a better PFS (19.4 months vs. 12.4 months, HR 0.59), although ORR were similar between the two arms [113].

In addition, erlotinib (combined with gemcitabine) is approved for the first-line and maintenance treatment of advanced pancreatic cancer, without targeting EGFR alteration [114].

Afatinib is a potent, selective, and irreversible inhibitor of the EGFR, HER2 and HER4 kinases. In advanced EGFR-positive NSCLC patients, afatinib showed a significant increase both in PFS and ORR, compared with platinum-based chemotherapy, in two large phase III trials: Lux-Lung 3 and Lux-Lung 6 [115,116]. Initially, OS rates were similar between the therapeutic options, but later, the combined analysis of these trials reported a statistically significant benefit in OS, but only in NSCLC with exon 19 deletion [117]. Regarding afatinib, the alterations G719X (exon 18 point mutation, 3–4% of all EGFR mutations), L861Q (exon 21 point mutation, 2% of all EGFR mutations) and S768I (exon 20 point mutation) are sensitive [118,119,120,121], while exon 20 insertions are resistant [122]. Afatinib was approved by the FDA (2013) for first-line treatment of EGFR-positive NSCLC (including the S768I, L861Q, G719X mutations).

Dacomitinib is an irreversible pan-HER inhibitor (HER1, HER2 and HER4). Compared with gefitinib, dacomitinib increased the PFS and OS rates in the ARCHER 1050 trial [123]. FDA approved dacomitinib for the first-line treatment of patients with metastatic NSCLC and EGFR exon 19 deletion or exon 21 L858R substitution.

The vast majority of NSCLC with EGFR sensitizing mutations eventually develop resistance to TKIs therapy, with a median time to progression (TTP) of approximately 10–12 months. In about 60% of cases, resistance is associated with a secondary T790M mutation in exon 20 of the EGFR gene. Other reported mechanisms of acquired resistance are: amplification of MET, HER2 and CRKL genes (20%), mutations in PI3K, KRAS or BRAF, activation of the AXL TKR, or histological transformation to small cell lung cancer (SCLC) [124,125,126,127]. Some of these molecular events are considered activating bypass tracks, because downstream signalling pathways are eventually stimulated, regardless of EGFR tyrosine kinase activity inhibition.

The previous survival results were surpassed with the arrival of osimertinib, a third generation, orally available, and irreversible TKI EGFR mutant-selective variants. Osimertinib inhibits a broad range of EGFR mutant forms, including the T790M. In a phase I/II study, osimertinib demonstrated a significant response rate of 61% and median PFS of 10 months in patients with NSCLC harboring a T790M mutation whose disease progressed on other EGFR-inhibiting therapy [128]. These favourable results allowed the FDA approval (2017) for the treatment of NSCLC after progression on after EGFR-TKI therapy. In the AURA3 trial, a randomized and phase III trial, osimertinib was compared with platinum-therapy plus pemetrexed, in the same setting of the disease. Osimertinib increased median PFS (10.1 months vs. 4.4 months, HR 0.3) and ORR (71% vs. 31%), and also demonstrated higher efficacy in central nervous system (CNS) disease [129,130].

The FLAURA trial (2019) tested osimertinib in the front-line treatment of EGFR-mutated advanced NSCLC (versus gefitinib or erlotinib). Newly, both median PFS and median OS were higher with osimertinib (18.9 months vs. 10.2 months, HR 0.46; 38.6 months vs. 31.8 months, HR 0.8, respectively). Disease control at CNS was ratified [131]. Due to FLAURA results, the FDA approved osimertinib as the standard therapy in the front-line setting, including EGFR-mutant NSCLC with uncommon mutations.

Some resistant mechanisms to osimertinib have been described, such as the KRAS amplification, the EGFR C797S mutation (which has not been seen in resistance to first-generation drugs) and the MET amplification [132,133].

TAS6417, tarloxotinib (TH-4000) and luminespib (Hsp90 inhibitor; AUY922) are novel inhibitors of EGFR, especially directed to exon 20 insertions. [134,135,136].

### 4.2. HER2

In breast cancer, HER2 overexpression is a poor prognostic factor, given the reported higher disease recurrence and death rates. It is considered a predictive factor for targeted therapy response, because the identification of high HER2 overexpression has allowed to detect patients who most benefit from these targetable drugs. At present, detection of HER2 overexpression is a standard in the diagnostic approach of breast cancer.

HER2 receptor activates through heterodimerization with other receptors, but external ligand binding is not necessary. This leads to activation of the following pathways, promoting cell survival and proliferation [137]. In this “HER2-blocking setting”, the approved TKIs by the FDA are lapatinib, neratinib and tucatinib.

Lapatinib is a dual and reversible TKI of EGFR and HER2, also with activity against Akt, Erk-1 and -2. Lapatinib has been tested in HER2-positive, both hormone receptor (HHRR)-positive and -negative metastatic breast cancer (MBC), and became the first TKI approved in this setting. In postmenopausal women with HER2-positive and HHRR-positive MBC (who had received no previous treatment with either trastuzumab nor aromatase inhibitors), the EGF30008 trial tested the combination of lapatinib plus letrozole (versus placebo plus letrozole) [138]. The combined treatment improved median PFS (progression risk was reduced by 29%), but not OS. These results supported the FDA approval. In addition, lapatinib has been combined with trastuzumab plus an aromatase-inhibitor (AI) in the ALTERNATIVE trial, which focused on postmenopausal women with HER2-positive and HHRR-positive MBC, but after receiving prior trastuzumab and endocrine therapy [139]. PFS and ORR were improved with the triplet (compared with trastuzumab plus an AI), but it remains unclear when is the most appropriate moment to add lapatinib in the therapeutic algorithm of those patients. This study has been recently retracted due to numerical corrections in some secondary efficacy analyses [140].

In the population with HER2-positive and HHRR-negative MBC, the EGF 104990 trial compared lapatinib plus trastuzumab against lapatinib alone, and the combination significantly improved the PFS (11 weeks vs. 8 weeks, HR 0.74) and the OS (14 months vs. 10 months, HR 0.74) in the population who had received one or more prior trastuzumab-containing regimens [141,142]. Nevertheless, its approval by the FDA (2007) includes the combination with capecitabine after progression on standard chemotherapy (anthracyclines and taxanes) and trastuzumab in the advanced disease setting. The benefit of this doublet was showed in a phase III trial of 399 patients with HER2-positive MBC, who were randomly assigned to receive lapatinib plus capecitabine versus capecitabine alone. PFS significantly improved with the combination (6 months vs. 4 months), but not OS (75 weeks vs. 65 weeks) [143,144].

Neratinib is an irreversible TKI of EGFR, HER-2 and HER-4. It locks on the cysteine residue in the ATP-binding pocket, to irreversibly inhibit the downstream intracellular pathways. First of all, in the adjuvant setting, one year of neratinib treatment demonstrated significant improvement of the 5-year invasive DFS rate (ExteNET trial) in women with HER2-positive breast cancer who had completed neoadjuvant and adjuvant chemotherapy plus trastuzumab, compared with placebo [145]. In 2017, this was the first FDA approval for neratinib, extending the adjuvant treatment after receiving trastuzumab-based therapy. Later, in the advanced setting, neratinib was approved, in combination with capecitabine, for the treatment of women with HER2-positive MBC who have received two or more previous regimens, based on the results of the NALA study [146]. This phase III trial randomized 621 patients to receive neratinib plus capecitabine versus lapatinib plus capecitabine. Twelve-months PFS rate was higher in the experimental arm (29% vs. 15%), although OS results were not statistically different (21 months vs. 18.7 months). Interestingly, neratinib has shown efficacy as a single agent in the metastatic setting (breast cancers carrying HER-2 LSS5 mutation) and combined with paclitaxel in a phase II trial [137,147]. Both lapatinib and neratinib are valid options (combined with capecitabine), even though efficacy data are higher with the second doublet [148,149].

Recently, tucatinib has emerged as a new TKI called to change the current therapeutic landscape of HER2-positive MBC. Tucatinib is an orally bioavailable inhibitor of HER-2, which selectively blocks the intracellular TK domain, exerting a minimal inhibition of EGFR. This distinct mechanism of action substantially modifies the side effects. In the initial phase I trial (2018), tucatinib plus capecitabine and trastuzumab demonstrate a promising response rate, even in a remarkable population with CNS metastases [150]. Afterwards, the HER2CLIMB trial (phase II, double-blinded and placebo-controlled) compared tucatinib plus capecitabine and trastuzumab against placebo plus capecitabine and trastuzumab) in heavily-pretreated women with HER2-positive MBC (median of four previous regimens) [136]. The triplet improved the 12-months PFS rate (33% vs. 12%, HR 0.54) and the 2-years OS rate (45% vs. 27%, HR 0.66) in the overall population, as well as in patients with CNS disease, whose 12-months PFS rate was clearly higher with the tucatinib-containing regimen (24.9% vs. 0%). In 2020, tucatinib has been approved, in combination with trastuzumab and capecitabine, for the treatment of advanced HER2-positive MBC after one or more prior HER2-based regimens.

Interestingly, HER2 mutations have been detected in lung cancer too (approximately 1 to 3% of NSCLC tumors). Insertions in exon 20 are the most common alteration [151,152]. In 2016, Mazières et al. published the results of a European cohort of 101 patients with NSCLC harboring HER2 mutations and treated with neratinib, afatinib and lapatinib (among other HER2-targeted therapies), setting a precedent for the treatment with TKI in HER2-positive lung cancer. However, data are not mature enough for its clinical use [153].

Poziotinib and pyrotinib are other HER2-targeted drugs currently under investigation in early phase trials [137,154,155].

The following table briefly summarizes the main EGFR and HER2 inhibitors developed and under development to date (Table 2).

### 4.3. Anaplastic Lymphoma Kinase (ALK)

The discovery of ALK involvement in cancer has changed the therapeutic approach of multiple neoplasms. In physiological circumstances, the ALK gen encodes for a transmembrane receptor with tyrosine kinase activity. This TKR belongs to the great insulin-receptor family. Its activation promotes receptor dimerization, which, in turn, will send the activating signal towards the following intracellular pathways: PI3K/Akt/mTOR, ERK, Stat3, RAS/MEK/Erb.

In solid tumors, alterations of ALK are especially involved in NSCLC oncogenesis, in such a dependent way that it has been called “addictive pathway”. Characteristically, ALK is altered through rearrangements that create fusion oncogenes (although also amplification and point mutations have been described) [156]. Such rearrangements involve the ALK gene loci on chromosome 2 and are found in 5–6% of NSCLC. The best-known rearrangement juxtaposes the 5′ end of the echinoderm microtubule-associated protein-like 4 (EML4) gene with the 3′ end of the ALK gene, resulting in the EML4-ALK fusion oncogene. Other gene partners can fuse with ALK: KIF5B, KLC1, TPR, HIP1, DCTN1, SQSTM1, NPM1, BCL11A, y BIRC6.

In the same way as EGFR, detection of ALK rearrangements is mandatory before starting any systemic therapy for advanced NSCLC, since it constitutes a predictive (dramatic) factor of response to TKI drugs. From a clinical point of view, ALK-positive NSCLC patients include never or light smokers, younger aged, and adenocarcinomas (with signet ring or acinar histology) [157,158].

There are several FDA-approved ALK-targeted TKIs: Crizotinib, ceritinib, alectinib, brigatinib and lorlatinib.

Crizotinib was the first ALK inhibitor that got the approval by the FDA (2011). It is a first-generation TKI, which selectively inhibits the tyrosine kinase domain and some oncogenic forms (including ALK fusions, such as with the NPM1 and BCL11A genes, and selected mutations). Likewise, crizotinib also inhibits the tyrosine kinase activity of c-MET (in fact, crizotinib was originally developed as a MET inhibitor), ROS1, Tropomyosine Receptor Kinase (TRK) and Recepteur d’Origine Nantais (RON). Initially, crizotinib was tested in patients with advanced ALK-positive NSCLC who has progressed on platinum-based chemotherapy, showing better 1-year PFS rate than chemotherapy [159]. Later, in the PROFILE 1014 phase II trial, crizotinib was compared against pemetrexed plus either cisplatin or carboplatin in the first-line setting [160]. Both median PFS and ORR were higher with crizotinib (10.9 months vs. 7 months, HR 0.45; 74% vs. 45%, respectively). Following analysis proved OS improvement after crossover adjustment with crizotinib over chemotherapy (HR 0.35) [161].

Despite these findings, novel ALK TKIs were developed and demonstrated better survival outcomes in the frontline treatment and after progression on crizotinib.

Ceritinib is a highly selective, second-generation TKI of ALK. It inhibits autophosphorylation of wild-type ALK, ALK fusion proteins and ALK point mutations. Ceritinib showed superiority over chemotherapy (platin plus pemetrexed) in the ASCEND-4 trial [162]. Both median PFS and ORR were better with ceritinib (16.6 months vs. 8.1 months, HR 0.55; 72.5% vs. 26.7%, respectively), including patients with CNS metastases. In patients without CNS disease, the PFS rates were even better (26.3 months vs. 8.3 months). Ceritinib was approved by the FDA in 2014 for the first-line treatment of advanced ALK-positive NSCLC.

Alectinib is another second-generation TKI of ALK, potent and highly selective against the tyrosine kinases of the receptor. It also blocks some ALK fusion proteins and the gatekeeper mutation L1196M (a known resistance mechanism) and RET (Rearranged during transfection). At first, the J-ALEX trial compared alectinib against crizotinib in a Japanese population with advanced ALK-positive NSCLC, who were chemotherapy-naïve or had received one previous chemotherapy regimen [163]. The median PFS was higher with alectinib. After that, the global study ALEX faced alectinib versus crizotinib in untreated ALK-positive NSCLC patients [164]. After a follow-up period of approximately 28 months, the PFS rate was significantly higher with alectinib (35 months vs. 11 months, HR 0.43). The HR for CNS progression was surprisingly better with alectinib (0.16) [165]. OS results are not yet mature. These data supported the FDA approval in 2015.

Secondary mutations arise as a resistance mechanism after exposure of tumor cells to ALK inhibitors, and most of them are within the ALK tyrosine kinase domain (“ALK-dependent mechanisms of resistance”), inducing the inability to inhibit the encoded tyrosine kinase. L1196M (the most common), G1269A, C1156Y, F1174L, 1151Tins, L1152R, S1206Y and V1180L are some of these acquired mutations that confer resistance to crizotinib [166]. This is particularly important, since in approximately 50% of cases, the CNS is the first site of disease progression. “ALK-independent resistance mechanisms” are EGFR, IGF-R1 or c-KIT mutations.

Both ceritinib and alectinib can be administered after progression on crizotinib. The ASCEND-5 study randomized 231 patients to receive ceritinib or chemotherapy [167]. All had advanced ALK-rearranged NSCLC and all had received prior chemotherapy and crizotinib, with disease progression after that. Ceritinib increased median PFS (5.4 months vs. 1.6 months, HR 0.49) and ORR (39.1% vs. 6.9%). In addition, in the ALUR trial, 107 patients with advanced ALK-positive NSCLC and previously treated with platinum-based chemotherapy and crizotinib were randomly assigned to alectinib or chemotherapy [168]. The median PFS was higher with alectinib (7.1 months vs. 1.6 months, HR 0.32). Two additional phase II trials support these results, and, also, reported rates of CNS disease control higher than 80% [169,170]

Brigatinib is a next-generation ALK inhibitor, whose anti-TK spectrum encompasses EGFR (mutant forms), ROS1, and Insulin Growth-Factor Receptor-1 (IGF-R1). Brigatinib prevents autophosphorylation of ALK and Stat3 downstream signalling pathway proteins. In preclinical studies, brigatinib demonstrated increased inhibitory capacity over some ALK resistance mutations, compared with crizotinib, ceritinib and alectinib (including G1202R, the most recalcitrant one, and L1196M), and also against non-ALK kinases [171]. In the first-line treatment of advanced ALK-positive NSCLC, brigatinib was approved (2017) based on the results of the phase III trial ALTA 1L. Two hundred seventy-five patients were randomly assigned to brigatinib or crizotinib, and both the 12-months PFS rate and the ORR among patients with brain metastases were higher with brigatinib (67% vs. 43%, HR 0.49; 78% vs. 26%, respectively) [172]. Subsequently, the ALTA trial provided evidence of brigatinib benefit in patient’s tumors refractory to crizotinib. In this phase II trial, 222 patients whose disease had progressed after crizotinib were assigned to receive different doses of brigatinib (90 mg vs. 180 mg), demonstrating better median PFS and median OS with the double dose (9.2 months vs. 16.7 months; 29.5 months vs. 34.1 months, respectively) [173]. Furthermore, the median response duration at CNS was higher with 180 mg (9.4 months vs. 16.6. months).

Finally, lorlatinib is a third-generation TKI of ALK, which competitively and selectively inhibits ALK kinases and the c-ROS oncogene. In preclinical studies, lorlatinib showed sustained inhibition of mutated and non-mutated variants of ALK, including the G1202R and I1171T resistant variants, which confer refractoriness to crizotinib, ceritinib, alectinib and brigatinib [174,175,176]. Lorlatinib clinical efficacy was demonstrated in a phase II study (B7461001), in which 198 patients with ALK-positive metastatic NSCLC previously treated with, at least, one ALK inhibitor, received lorlatinib [177]. After crizotinib, the ORR with lorlatinib reached 40%, with a median PFS of 11.1 months; after one or two second-generation ALK TKIs, ORR was higher among patients with ALK mutations [178]. Taking these results into account, the FDA approved lorlatinib in 2018 for the treatment of advanced ALK-positive NSCLC which has progressed on crizotinib and, at least, another ALK TKI; or, after a second-generation ALK TKI in the frontline setting.

### 4.4. ROS1

*c-ROS* is another proto-oncogene which encodes a transmembrane type-1 receptor with tyrosine kinase activity, very structurally similar to the ALK receptor. It is a member of the insulin-receptor family (without clearly identified ligands) and its activation generates signalling cascades whose objective remains unclear. Naturally, it is highly expressed in kidney, cerebellum, small bowel and colon.

Some molecular alterations have gained attention in the last years, specially ROS1 fusions, present in 2–3% of the lung adenocarcinomas, as well as in cholangio, gastric and colonic carcinomas [179]. They are present in spitzoid neoplasms, glioblastoma multiforme and inflammatory myofibroblastic tumors, too. Approximately 30 genes have been identified as ROS1 fusion partners, some of them were also known for being ALK and RET fusion partners as well. The acquisition of these fusion genes induces the constitutive activation of the tyrosine kinase activity of the receptor, which in term deregulates cellular processes as differentiation, proliferation and survival. Genes most frequently described as ROS1 fusion partners in NSCLC are CD74 (most frequent), SLC34A2, TPM3, SDC4 and EZR [180]. Interestingly, every ROS1 fusion partner activates different signalling pathways depending on the heterodimeric partner. However, the mutations in ROS1 can constitute a resistance mechanism in patients treated with crizotinib as first-line treatment of ALK-rearranged NSCLC, as shown by G2032R mutation [181]. Typically, ROS1 fusion NSCLCs are present in women without known tobacco consumption.

For diagnostic purposes, ROS1 fusions are mutually-exclusive with other driver mutations.

Crizotinib is one of the two drugs approved for the treatment of tumors with ROS1 rearrangements. Shaw et al., published the results of crizotinib in a cohort of 50 patients with ROS1-rearranged NSCLC, showing an ORR of 72% and a median PFS of 19.2 months [182]. No correlation between the type of ROS1 rearrangement and response to crizotinib was found. Later, update on survival rates from the PROFILE 1001 trial confirmed the previous response rate and showed a median duration of response of 24.7 months and a median OS of 51.4 months [183]. In 2016, crizotinib was approved for the treatment of advanced ROS1-positive NSCLC patients, who were treatment-naïve and those who had received previous chemotherapy.

Entrectinib is a pan-TRK inhibitor with affinity against ROS1 and ALK too. This drug is approved for the treatment of ROS-1 rearranged NSCLC because of the results of the pooled analysis of the ALKA-372-001, STARTRK-1 y STARTRK-2 trials [184]. In these trials, treatment with Entrectinib in patients with locally advanced or metastatic ROS1-fusion positive NSCLC offered a benefit in terms of a 77% of ORR, a median duration of response of 26.6 months and a median PFS of 19 months. Due to its high intracranial penetration, entrectinib is preferred for treatment of advanced disease with CNS metastases.

Given the structural homology between ALK and ROS1, ceritinib, brigatinib and lorlatinib show promising activity against ROS1 fusions. Ceritinib showed near to 62% of response rates and a median PFS of 9.3 months in a phase II clinical trial [185]. In patients who have progressed to crizotinib, there is data from a phase I/II trial favoring the use of lorlatinib, with a 35% response rates [186].

As a future view, repotrectinib is a new TKI designed to potently inhibit some secondary and resistant mutations of ALK, ROS1 and TRKA-C (for example, D2033N in ROS1, G595R in TRKA and G623R in TRKC) [187]. Its antitumoral activity has been proven in preclinical and cells models.

### 4.5. Neurotropic Tropomyosine Receptor Kinase (NTRK)

NTRK 1, 2 and 3 genes encode three proteins called TRKA, TRKB and TRKC, respectively. These proteins are TKR involved in neural development. Each one of them contains a ligand-binding extracellular domain, a transmembrane region and a unique intracellular kinase domain. Each receptor has its preferential ligand: TRKA with neurotrophin nerve growth factor (NNGF), TRKB with brain-derived neurotrophic factor (BDNF) and neurotrophin-4, and TRKC with neurotrophin-3.

TRKA, TRKB and TRKC have been identified as oncogenic drivers in a variety of malignancies. The most frequent pathogenic mechanism described are rearrangements [188]. Mutations or gene amplifications have been described, but seem to have no role in carcinogenesis. Rearrangements usually occur between the C-terminal end of the tyrosine kinase domain with the N-terminal end of the fusion partner, and this is usually secondary to chromosomic inversions, deletions or translocations. The fused protein has no ligand-binding domain, and dimerisation and phosporylation ocurr in an independent manner.

NTRK fusions characteristically appear in rare tumors, like secretory carcinoma of the breast, fibrosarcoma infantile and salivary gland carcinoma. In more frequent tumors like NSCLC, colorectal and thyroid cancer, melanoma or gliomas, the prevalence of these alterations is very low [189]. For example, in NSCLC, it is approximately 1%.

Larotrectinib and entrectinib are the only drugs approved by the FDA for the treatment of advanced solid tumors with NTRK fusions, and are approved for previously treated patients. Efficacy of larotrectinib was demonstrated in various phase I and II clinical trials. Hong et al. reported the first data of anti-tumoral activity in 70 patients with harboring NTRK gene fusions [190]; Later, Drilon et al. published the results of the phase I/II trial [191]. Larotrectinib showed an ORR of 75%; with a median follow-up of 9.4 months, 86% of the patients continued treatment with the drug. The 12-months PFS rate was 55%. The largest and most recent publication of larotrectinib focused on its long-term efficacy and safety. Included 159 patients with multiple types of neoplasms, reporting ORRs of 79% (including 16% of complete responses), concluding that NTRK rearranged tumors are highly sensitive to larotrectinib [192].

Entrectinib was also approved in base of the combined results of various early-phase trials (ALKA-372-001, STARTRK-1 y STARTRK-2). As we have discussed before, entrectinib is able to inhibit TRKA, TRKB y TRKC. Its activity in solid tumors harboring NTRK fusions was evaluated in 54 patients, reaching an ORR of 57% (including 7.4% of complete responses) and a median duration of response of 10 months [193].

Cabozantinib minimally inhibits TRK, specifically TRKB, but it has not been approved in this setting. Crizotinib, ponatinib and nintedanib show different affinity against TRK.

Resistance mechanisms to TRK inhibitors published so far essentially lie in secondary mutations of the kinase domain (G595R, A608D and G667S in TRKA, G623R and G696A in TRKC). LOXO-195, TPX-0005 and ONO-539055 are next-generation TRK inhibitors developed to potentially overcome these acquired resistances, showing promising activity in in vitro assays [188,194].

The following table briefly summarizes the main ALK, ROS1 and NTRK inhibitors developed and under development to date (Table 3).

### 4.6. Fibroblast Growth Factor Receptor (FGFR)

Four highly conserved transmembrane TKR form the FGFR family, corresponding to the FGFR 1, 2, 3, and 4. FGFR dimerization precedes the phosphorylation signal of the tyrosine kinase domain, which, in turn, leads to signal transduction (RAS/RAF/MAPK, PI3K/Akt/mTOR, etc.). In non-cancer cells, FGFR is involved in intracellular signaling cascades that regulate functions of homeostasis, embryonic development, endocrine and repair functions.

FGFR dysregulation has been related to oncogenic processes, especially with tumor progression and resistance mechanisms to classical chemotherapy. FGFR aberrations have been described in many tumors (including receptor amplification, mutations, and chromosomal translocations) [195]. FGFR1 amplifications have been found in squamous NSCLC, in SCLC, and in MBC (15% of the HHRR-positive, 5% of the triple-negative subtype). FGFR2 amplifications have been discovered in 5-10% of gastric cancers and in 4% of triple-negative breast cancer. FGFR mutations are more common in FGFR2 and FGFR3, predominantly occurring in the ligand-binding and transmembrane domains of the receptor. The former is found in 10-12% of endometrial carcinomas, in 4% of NSCLC and gastric cancers, and in 2% of urothelial cancers. FGFR3 mutations are found in approximately 75% of non-muscle invasive urothelial carcinomas and in 15% of high-grade urothelial carcinomas. Activating gene fusions have also been described: involving FGFR3, especially in glioblastoma and bladder cancer, and FGFR2, in approximately 15% of cholangiocarcinomas (and, less commonly, in lung, thyroid, and prostate cancers).

Currently, many drugs have multikinase inhibitory activity and partially block FGFR, but only two specifically targeted TKIs against FGFR have been approved by the FDA for clinical use: erdafitinib and pemigatinib.

Erdafitinib is a pan-FGFR inhibitor. It binds to the receptor, inhibits its phosphorylation and avoids the signal transduction. Erdafitinib demonstrated clinical efficacy in the BCL2001 study [196]. This open-label and nonrandomized phase II trial tested erdafitinib in 99 patients with advanced urothelial carcinoma and known FGFR3 mutations and FGFR2/FGFR3 fusions. Previous immunotherapy was allowed. The ORR was 40% (59% in the immunotherapy-treated subgroup) and the median PFS was 5.5 months (13.8 months in the immunotherapy-treated subgroup). Given these positive results, in 2019 erdafitinib became the first-ever FGFR kinase inhibitor approved for the treatment of patients with advanced or metastatic urothelial carcinoma with susceptible FGFR2 or FGFR3 genetic alterations, after progression on platinum-based chemotherapy.

Pemigatinib is a selective inhibitor of the FGFR types 1, 2 and 3 so, subsequently, inactivates the related signal transduction pathways. Its safety and antitumor activity were tested in the single-arm FIGHT-202 trial, which included patients with advanced cholangiocarcinoma who had progressed to, at least, one previous line of systemic treatment. FGFR2 fusions or rearrangements, other FGFR abnormalities, and FGFR normal status were included [197]. Surprisingly, among the population with FGFR2 fusions or rearrangements, 36% of patients responded to treatment and 80% achieved, at least, stable disease. In the population with other FGF/FGFR alterations, 40% of patients reached stable disease. Because of these results, in April 2020, the FDA approved pemigatinib for the treatment of patients with unresectable locally advanced or metastatic cholangiocarcinoma previously treated, and with FGFR2 gene fusion or rearrangements.

Others multi-kinase inhibitors, such as lenvatinib, nintedanib, pazopanib, ponatinib, and regorafenib target FGFR with variable affinity, but they have no indication for FGFR-driven treatment.

The following table briefly summarizes the main FGFR inhibitors developed and under development to date (Table 4).

### 4.7. c-KIT

*c-Kit* is a proto-oncogene present on chromosome 4, which encodes a receptor with tyrosine kinase activity (KIT receptor). Its ligand is the Stem Cell Factor (SCF). KIT has three domains: one extracellular, for the ligand binding, one transmembrane, and one cytoplasmic with the tyrosine kinase function. Physiologically, binding of the SCF ligand allows the dimerization of two KIT receptors and their autophosphorylation, to initiate downstream signalling.

KIT is physiologically involved in fertility processes, intracellular homeostasis, differentiation of hematopoietic cells, melanogenesis and development of other cell lines (erythrocytes, mast cells, interstitial cells of Cajal, and sweat glands cells). Its oncogenic role in gastrointestinal stromal tumors (GIST) was studied in 1998 by Hirota and colleagues, demonstrating that KIT activating mutations occurred in the absence of natural ligand [198]. In approximately 80–85%, the KIT receptor is overexpressed, and this aberration is associated with an activating mutation of the c-Kit protooncogen [199]. This finding marked a milestone in the history of molecular medicine and set the basis for targeted therapies.

It is estimated that 80–85% of GISTs show some c-Kit activating mutations. Additional c-Kit alterations have also been described in various neoplasms: leukemias, melanoma, germ cell carcinomas, adenoid cystic carcinomas, and other human cancers.

As we mentioned, the gain activity of c-Kit has been associated with activating mutations (mostly) and overexpression. The main mutations of c-Kit (in GIST) are present in exons 11 (65%), 9 (15%), 17 (2%) and 13 (2%), and they encode distinct molecular regions of the receptor (juxtamembrane domain, extracellular dimerization domain, activation loop domain, TKI and ATP-binding pocket, respectively) [199]. However, not all c-Kit mutations are activating. For instance, c-Kit activating mutations in melanoma induce apoptosis. Characteristically, each neoplasm presents its own c-Kit mutations, which may vary in the location of the exon and in the type of mutation. It is well known that those tumors harboring a c-Kit mutation and, as a consequence, boost the TKR function, are those which can benefit from the targeted therapy.

Imatinib is a potent antineoplastic agent which inhibits the tyrosine-kinase activity of the Bcr-Abl fusion protein, KIT, Platelet-Derived Growth Factor Receptor Alpha (PDGFRA), PDGFR Beta (PDGFRB), DDR1, DDR2 and CSF-1R. Its structure mimics ATP, so it competitively joins to the c-Kit binding site, avoiding phosphorylation of the substrate and the consequent signalling cascade. Imatinib administration in patients with GIST led to a dramatic, rapid and sustained clinical response in tumors that, historically, were considered “chemo-resistant”. The first trial to test imatinib in advanced GIST was published by Demetri et al. It was a phase II trial which randomly assigned 147 patients to receive either 400 mg or 600 mg of imatinib mesylate [200]. In 81.6% of the overall population, imatinib achieved disease control (53.7% of objective response plus 27.9% of stable disease). Imatinib was approved in 2001 for the treatment of advanced or unresectable GIST. A long-term analysis revealed that 18% of patients were still receiving imatinib after 9.4 years of follow-up [201]. At the 2014 annual meeting of the American Society of Clinical Oncology (ASCO), a preliminary analysis of the SWOG S0033 trial showed that 26% of patients survived 8 years or more, and the estimated 10-year survival rate was approximately 22% [202].

Proliferation and survival GIST cells are TKR-dependent processes, that is the reason why these tumors are commonly denominated “KIT addicted”. This phenomenon explains the need for continuous treatment with imatinib. In this regard, dose increasing has not obtained a clear benefit in OS [203,204]. A meta-analysis of some studies showed that the presence of an exon 9 mutation was the only significant predictive factor for benefiting from a higher dose, as the better PFS and OR rates were reported with 800 mg daily [205].

In the locally advanced setting of GIST, imatinib is approved for the adjuvant treatment of resected GIST with a high risk of relapse [206].

The vast majority of patients improves with imatinib. However, 5% of patients have primary resistance to imatinib 400 mg, and 10% will develop early resistance to the treatment, defined as progression in the first 6 months of therapy [206]. One the one hand, these patients probably do not have mutations in c-Kit (in that case, within exon 9) or in PDGFRA genes. On the other hand, the acquisition of resistance to imatinib is basically related to secondary mutations in the c-Kit gen, an event that usually occurs two years after the treatment starts [203,207]. These resistance mechanisms can be divided into: KIT primary mutations, KIT ATP-binding pocket secondary mutations, KIT activation loop secondary mutations, and acquired loss of KIT oncoprotein expression.

Sunitinib and regorafenib are multikinase inhibitory drugs (detailed in the “Vascular Endothelial Growth Factor Receptor” section), and both are approved for the treatment of advanced GIST after progression to imatinib. Sunitinib (compared with placebo) showed clinical activity in patients with refractory GIST in a phase III trial: median PFS was higher with sunitinib (27 weeks vs. 6 weeks), but not the median OS [207]. There appears to be a greater clinical benefit in patients with the KIT exon 9 mutation and in patients without the KIT or PDGFRA mutation. The above results allowed its approval by the FDA in 2009. Later, the FDA approved regorafenib for the treatment of advanced GIST refractory to imatinib and sunitinib. This was based on the results of the phase III trial GRID, of 199 patients who progressed on sunitinib: compared to placebo, regorafenib increased median PFS (4.8 months vs. 0.9 months, HR 0.27), with no apparent improvement in OS [208].

Serrano et al. published (in cell cultures) the variety of acquired resistances in the KIT gen as a mechanism of resistance to imatinib [209]. In their work, they observed that V654A (exon 13) and T670I (exon 14) mutations were more sensible to sunitinib, while activating loop mutations D816E and D820A (exon 17), especially when the primary mutation occurred in exon 11, were more sensible to regorafenib. Head to head comparisons between sunitinib and regorafenib have demonstrated that sunitinib was more active to block KIT exon 13 V654A ATP-binding pocket mutant.

Several KIT TKIs are currently under research and some of them have already provided significant results of safety and efficacy (for example, nilotinib, dasatinib, sorafenib, and pazopanib) [210].

### 4.8. Platelet-Derived Growth Factor Receptor (PDGFR)

PDGFR is another receptor with tyrosine kinase activity, whose initial researches were born from a better understanding of the VEGFR functioning. The PDGF constitute a set of polypeptide growth factors (called A, B, C and D), and which are fundamentally involved in the natural process of angiogenesis, although they also enhance the growth of fibroblasts, smooth muscle cells and glial cells derived from platelets. Its main receptor is PDGFR. There are two different types of class III receptors: Alpha (PDGFRA) and Beta (PDGFRB). Numerous studies have revealed the close correlation between PDGF and VEGF: most PDGF proteins play an important role in angiogenesis, directly stimulating endothelial cell proliferation and VEGF secretion. Apparently, the most important function of PDGFB is to recruit perivascular cells during angiogenesis.

At the tumor level, PDGF is known to take part in autocrine stimulation of cancer cells, angiogenesis stimulation, and control of interstitial cells growth.

In vitro, constitutive phosphorylation of PDGFRA has been shown to exhibit ligand-independent kinase activity [211]. Furthermore, the following signalling pattern is very similar to that activated by KIT.

Six to eight percent of GIST show activating mutations of PDGFRA [212]. The most frequently mutated exons are 18 (82.5%), 12 (13.7%), and 14 (3.7%) [213]. Exon 18 encodes the activation loop in the second tyrosine kinase domain. As we mentioned before, imatinib preserves inhibitory activity against PDGFR, but sensitivity to imatinib in PDGFR-mutated GIST is variable. The intrinsic resistance of the D842V mutation (substitution) in exon 18 is relevant, which is the most common (62%) and shows negative response rates: 68% disease progression in the largest study. Other exon 18 mutations (D846Y, N848K, Y849K) are sensitive to imatinib [214,215]. In conclusion, imatinib is still the initial therapy for patients with PDGFRA mutant GIST.

In addition to GIST, imatinib is approved for the treatment of recurrent, unresectable, or metastatic dermatofibrosarcoma protuberans (DFSP). Almost all cases of DFSP have the peculiar chromosomal translocation t(17:22), which subjects control of PDGFB to the collagen type I Alpha 1 (COL1A1) promoter [216,217]. This change finally results in constitutive activation of the PDGFB. Several reported clinical cases, two prospective trials and a systematic review provided high efficacy of imatinib in unresectable locally advanced or metastatic DFSP (and in the neoadjuvant setting, where some clinical trials have been published) [218,219,220,221,222].

Avapritinib (BLU-285) is a potent and selective TKI of KIT and PDGFRA, specifically targeting to some mutations at exons 11 and 17 of KIT, as well as D842 mutations of PDGFR. It is considered the gold-standard treatment for advanced GIST with D842V mutation. Different doses of daily avapritinib (300 mg and 400 mg) were tested in the phase I trial NAVIGATOR, which included 56 patients with advanced D842V-mutated GIST [223]. The reported response rate was 88% and the 2-years OS rate was 81%. These data justified the FDA approval in January 2020, indicating avapritinib for adults with metastatic GIST harboring a PDGFRA exon 18 mutation (including D842V mutations).

Ripretinib is a switch dual TKI that inhibits both wild-type and mutant forms of KIT and PDGFRA, avoiding the switch from inactive to active conformations. The activity range of ripretinib encompasses exons 9, 11, 13, 14, 17 and 18 mutations (in c-Kit), as well as exons 17 and 18 mutations (in PDGFRA). Ripretinib also targets the PDGFRA D842V and D816V resistance mutations, and inhibits other molecules, such as PDGFRB, VEGFR2, TIE2, and BRAF. In the INVICTUS trial, 129 patients with advanced GIST who had progressed to imatinib, sunitinib and regorafenib, were randomly assigned to receive ripretinib or placebo [224]. Ripretinib improved median PFS (6 months vs. 1 month, HR 0.15), ORR (9% vs. 0%) and median OS (15 months vs. 6 months, HR 0.36). These impressive results allowed its approval by the FDA in May 2020 for the treatment of advanced GIST after progression to 3 or more previous TKIs (including imatinib).

In addition to the drugs previously mentioned, many other TKIs target PDGFR. Most share VEGFR inhibition, so they are discussed in the “Vascular Endothelial Growth Factor Receptor” section.

The following table briefly summarizes the main c-KIT and PDGFR inhibitors developed and under development to date (Table 5).

### 4.9. c-MET

MET or hepatocyte growth factor receptor (HGFR) is a TKR distributed along multiple epithelial subtypes of the organism, and it is essential for embryonic development and tissue repair. Its main ligand is the Hepatocyte Growth Factor (HGF). In physiologic conditions, MET forms a heterodimer consisting of an external alpha subunit, a transmembrane beta subunit, and four immunoglobulin-like domains. The intracellular region of MET contains tyrosine kinase activity. MET has its own downregulation activity, whose protein sequence (juxtamembrane region) is encoded in exon 14 [225]. Skipping mutations in these loci are characteristic of NSCLC (3% of adenocarcinomas and 20% of sarcomatous carcinomas) and, molecularly, lead to excessive receptor activation due to reduction of MET protein degradation (the aim of the juxtamembrane region), promoting a sustained activation of paracrine and autocrine signals. This event has been associated with a worse prognosis [226]. Amplifications of c-MET and its receptor overexpression have also been described. Such alterations generate overactivation of various intracellular signalling pathways, such as Stat3, PI3K/Akt, mTOR, and RAS/MAPK [227].

Several TKI block MET, but only capmatinib specifically targets this substrate. Capmatinib is an oral TKI which selectively blocks the c-MET protooncogene, preventing both receptor phosphorylation and signal transduction. The GEOMETRY-mono-1 clinical trial tested capmatinib in 97 patients with advanced MET-exon-14-skipping mutated NSCLC, reaching an impressive ORR of 68% and a median PFS of 9.7 months [228]. In the overall capmatinib-treated population, ORR and median PFS were 41% and 5.4 months, respectively.

Other studies have reported favorable results with capmatinib in this setting [229]. With these data, capmatinib obtained the FDA approval in May 2020 for the treatment of patients with advanced NSCLC and a MET exon-14-skipping mutation.

Crizotinib, as explained above, is able to inhibit MET. Some studies have tested crizotinib in patients with MET exon-14-altered NSCLC, including the skipping-mutated population, with good results [230,231,232]. In 2018, the FDA granted Breakthrough Therapy designation for crizotinib for the treatment of patients with metastatic NSCLC with exon 14 alterations and disease progression on or after platinum-based chemotherapy.

Cabozantinib (multi-TKI), tepotinib (MET-selective TKI), glesatinib (multi-TKI) and savolitinib (MET-selective TKI) are other MET inhibitors that are under research in clinical trials [233,234].

The following table briefly summarizes the main MET inhibitors developed and under development to date (Table 6).

### 4.10. RET

*RET* oncogene was identified from studies of T-cell lymphomas DNA. This chimeric gene (due to recombination of unlinked DNA sequences) encodes a transmembrane fusion protein with tyrosine kinase intracellular domain characteristically ligated to a cadherine domain in the extracellular region. Its natural ligands are members of the glial cell-line derived neurotrophic factor (GDNF) family, such as GDNF, neurturin, artemin, and persephin.

Oncogenic activity of RET was first described on papillary thyroid carcinomas (PTC). RET rearrangements and the proto-oncogen PTC are present in 5–40% of the cases [235]. This oncogenic mechanism seems to be dependent on large somatic rearrangements with a variety of activating genes, which in turn allows the constitutive dimerization of the chimeric proteins RET/PTC. In addition, point mutations in RET are responsible for the constitutive activation of its tyrosine kinase activity in inherited MEN-2 syndrome, and in 30–50% of patients with sporadic medullary thyroid carcinoma (MTC) [236,237,238].

Selpercatinib is a selective RET inhibitor. It also targets VEGFR1, VEGFR3 and FGFR1-3 (although it is more specific for RET). Multicohort, phase I/II LIBRETO-001 trial allowed the approval of selpercatinib in RET-rearranged NSCLC and in advanced RET-mutant MTC and RET fusion-positive thyroid cancer which require systemic therapy [239,240,241,242]. One hundred and forty-three patients with MTC, treatment-naïve or previously treated with cabozantinib and/or vandetanib, received selpercatinib, reaching an ORR of approximately 70% and a 12-months PFS rate superior to 80%. In RET-fused previously treated differentiated thyroid carcinomas (DTC) (*N* = 19), the ORR was of 79%. In regard to NSCLC, 39 treatment-naïve patients with RET-rearranged NSCLC achieved an ORR of 85%, including those with CNS metastatic disease. RET rearrangements have been detected in nearly 1–2% of lung adenocarcinomas. The confirmatory phase III trials currently undergoing are the LIBRETTO-431 and LIBRETTO-531.

Other RET-specific TKIs, like pralsetinib (BLU-667, which selectively inhibits RET), are being tested in the phase I dose-escalation and phase II expansion ARROW trial, with promising results, showing an ORR of 73% in RET-mutant MTC patients and an ORR of 91% in 11 patients with RET-fused DTC [243]. Interestingly, the TPX-0046, a potent and selective next-generation TKI with activity against SRC kinase, has emerged as a targeted agent to potentially overcome resistance to first-generation selective RET inhibitors [244,245].

Alectinib, cabozantinib, lenvatinib, ponatinib, regorafenib, sorafenib, sunitinib and vandetanib are other potent TKI with nonselective RET inhibitory activity, but have been used to block this target. At present, sorafenib and lenvatinib are approved in radioiodine refractory DTC [246,247]. Cabozantinib and vandetanib are approved for the treatment of metastatic MTC [248,249].

The following table briefly summarizes the main RET inhibitors developed and under development to date (Table 7).

### 4.11. Vascular Endothelial Growth Factor Receptor (VEGFR)

VEGFR represent a family of three receptors, named VEGRF1, VEGFR2 and VEGFR3, being the most important from a biological point of view VEGFR2, as it is mainly expressed in the vascular endothelium. As we have discussed in previous paragraphs, VEGFR regulates the development of new blood vessels, in a process called angiogenesis, and in the regulation of its permeability, as we have discussed before [91,92,93]. This group of tyrosine kinase inhibitors are commonly called as antiangiogenics.

The structure of the receptor consists of seven immunoglobulin-like domains, which form the extracellular part of the receptor; the transmembrane domain and the intracellular tyrosine kinase domain. The receptor can be activated by different ligands, such as VEGF A/B/C/D, placenta growth factor (PlGF), parapoxvirus VEGFE, snake venom VEGFF and neuropilins NRP1 and NRP2 [250] or by mechanical stimulus coming from the environment, in a process called mechanotransduction [251]. This leads to the activation of PI3K/AKT/mTOR pathway, GTPases like RHO and RAC1 and eNOS1, regulating cell proliferation, survival, motility and vascular permeability [60,93,252].

It is particularly important to point out that most of the drugs listed below are multi-TKI with affinity to one or more TKRs. The drugs that are currently approved are listed below:

Sunitinib is an inhibitor of PDGFR (α and β), VEGFR (1, 2 and 3), KIT, Fms-like tyrosine kinase 3 (FLT3), CSF-1R and RET. Approved by FDA and EMA for the treatment of advanced GIST (as we cited before), advanced renal cell carcinoma (mRCC) and progressive, well-differentiated pancreatic neuroendocrine tumors (pNET). In these indications, sunitinib significantly prolonged TTP and OS in imatinib-refractory GIST [208], PFS, ORR and OS (patients who crossed over from IFN-α to sunitinib were censored) in mRCC, compared with IFN-α [253]; and PFS in pNETs in comparison to placebo [254].

Pazopanib shows activity against VEGFR (1-3), PDGFR (α and β), FGFR and cKIT. Approved by FDA and EMA for the treatment of mRCC and soft-tissue sarcoma who have received prior chemotherapy. This decision was based on phase III trials that demonstrated superiority against placebo and non-inferiority against sunitinib [255,256]. In soft tissue sarcoma, approval was based on the PALLETTE study, which included many different types of soft tissue sarcoma except adypocitic sarcoma, chondro and osteosarcoma, and DFSP [257].

Tivozanib is a TKI which selectively inhibits VEGFR1-3 at picomolar concentrations. Approved by EMA for the treatment of mRCC which are VEGFR and mTOR inhibitors-naïve and after progression to cytokine therapy. This approval was based on the TIVO-1 trial, with benefit in PFS, but not in OS [258].

Axitinib is another selective inhibitor of VEGFR 1-3. Approved by FDA and EMA for the treatment of mRCC, as monotherapy after progression to a prior treatment based on the AXIS trial in which a benefit in PFS was observed against sorafenib but did not improve OS [259]. In the first-line setting, axitinib is approved in combination with pembrolizumab based on the results of the MK426 trial [260] or with avelumab based on the results of the JAVELIN Renal 100 trial [261]

Cabozantinib is able to inhibit c-MET, VEGFR2, RET, TRKA, TRKB and Axl. Cabozantinib is approved by the FDA and EMA for the treatment of intermediate and poor risk mRCC in the first-line setting based on the CABOSUN phase II trial [262], and in all risk groups after progression to another TKI based on the METEOR phase III trial [263]; in hepatocellular carcinoma after treatment with sorafenib, under the Cabometyx*^®^* brand [264]; and progressive, unresectable locally advanced or metastatic MTC under the Cometriq*^®^* brand [248]. They are not interchangeable as the formulation is not equal. Cabometyx*^®^* comes as a tablet and Cometriq*^®^* as a capsule.

Lenvatinib is an inhibitor of VEGFR (1–3), FGFR (1–4), PDGFRα, c-KIT and RET. Approved by FDA and EMA for the treatment of patients with locally recurrent or metastatic, progressive, radioactive iodine-refractory DTC [265]; in combination with everolimus, for the treatment of patients with mRCC following one prior anti-angiogenic therapy [266] and for the first-line treatment of patients with unresectable hepatocellular carcinoma [267].

Sorafenib shows activity against VEGFR (1–3), PDGFRβ, Flt-3, c-KIT, RET and Raf. Inhibition of Raf has apoptotic effect in tumoral cells [268,269]. Sorafenib was the first systemic treatment to be approved for the treatment of advanced hepatocellular carcinoma, based on the SHARP study [270]. It is also approved for locally recurrent or metastatic, progressive, DTC refractory to radioactive iodine treatment; with benefit on PFS but not in OS and for the treatment of good and intermediate-risk mRCC after progression to cytokine treatment and [246,271].

Regorafenib: The structure of this compound is similar to sorafenib, but it differs in the addition of a fluorine atom in the proximal phenyl ring. The activity is similar to Sorafenib being able to inhibit Raf, TIE-2, PDGFR, VEGFR, RET and CSF-1R [272,273] A Approved by the FDA and EMA for the treatment of patients with metastatic colorectal cancer who have been previously treated with, or are not considered candidates for available therapies, which include fluoropyrimidine-based chemotherapy, an anti-VEGF therapy and an anti-EGFR therapy [274]; locally advanced, unresectable or metastatic gastrointestinal stromal tumor who have been previously treated with imatinib mesylate and sunitinib malate, offering benefit in PFS but not in OS [208]; and hepatocellular carcinoma who have been previously treated with sorafenib [275].

Nintedanib: This drug shows activity against VEGFR (1-3), PDGFR (α and β), FGFR (1-3), TRK, Flt-3 and RET. Approved by the EMA for the treatment of NSCLC with adenocarcinoma histology, locally advanced, metastatic of locally recurrent after first line-chemotherapy, based on the result of the LUME-Lung-1 trial, with modest benefit in PFS and OS in combination with Docetaxel [276].

Vandetanib: Inhibitor of VEGFR, EGFR and RET. Vandetanib is approved by the FDA and EMA for the treatment of symptomatic or progressive MTC patients with unresectable locally advanced or metastatic disease [249].

Other antiangiogenics that deserve our attention are apatinib (also known as rivoceranib), fruquintinib and anlotinib. Apatinib is approved for the treatment of advanced gastric carcinoma in China based on the results of a phase II trial [277]. The results of a phase III with rivoceranib failed to show benefit in OS but the result of secondary endpoints as PFS were positive, and the drug is under FDA evaluation for approval [278]. In a similar way, fruquintinib is approved in China for the treatment of advanced colorectal cancer and anlotinib for the treatment of non-squamous cell lung cancer [279,280].

The following table briefly summarizes the main antiangiogenics developed (Table 8) and under development to date (Table 9). Another table on the main antiangiogenics and their affinity for each receptor is presented in Appendix B (Table A2).

## 5. Discussion

The development of TKI against different TKR has become an important part of progress in the landscape of cancer treatment and has opened a stimulating field of research in precision oncology. The increasing availability of genomic, transcriptomic, proteomic and metabolomic data has offered us a broad vision of the heterogeneity and complexity of an individual tumor, driving us to an era of molecular-based therapy over a histology-based one [281].

There is a growing tendency to perform a tumor-agnostic strategy to treat cancer. This consists on initial screening with next-generation sequencing (NGS) to detect multiple potential targets for specific molecules. We can distinguish between basket and umbrella trials: In the former, patients are selected based on the same molecular alteration in spite of the tumor origin, whilst in the latter, they are selected based on the same tumor type or location and then subdivided depending on the molecular alteration identified. The tumor-agnostic strategy allows us to offer a gene alteration-targeted therapy in different kind of tumors or diverse therapies within the same histology. One of the main limitations is that the same driver mutation may require different therapeutic approaches among diverse cancer types because of the specificities in resistance mechanisms. It is important to underline the complexity of designing this type of studies, the high costs involved, the difficulty in recruiting patients and the accomplishment of regulatory agencies requirements for the drug approval [281,282,283].

One example of a basket trial is the NCI-MATCH (the NCI’s Molecular Analysis for Therapy Choice): a non-randomized phase II study for advanced refractory solid tumors, lymphomas, or multiple myeloma who have progressed to previous treatment. They are subdivided into 37 substudies depending on the identified druggable mutation, showing interesting preliminary results in terms of ORR. Another interesting basket trial is the NCI-MPACT (the NCI’s Molecular Profiling-Based Assignment of Cancer Therapy), a phase II study for advanced refractory solid tumors which are tested for 20 genes belonging to three molecular pathways broadly known to be involved in tumorigenesis -MAPK, PI3K and DNA repair- and then randomized 2:1 to different therapies depending on the mutation found. No results have been posted to date (NCT01827384) [283,284].

The idea of giving the right treatment for the right patient at the right time has still a long way ahead to be validated by positive results in the basket or umbrella-designed trials due to the limitations presented above. To date, there are 3 FDA-approved tumor-agnostic therapies: pembrolizumab in the case of microsatellite instability-high (MSI-H) or deficient mismatch repair (dMMR) and, very recently, also for mutational burden-high (TMB-H) (≥10 mutations/megabase (mut/Mb)) solid tumors and larotrectinib or entrectinib for patients with unresectable or metastatic solid tumors with NTRK fusion. Other candidates are selpercatinib or pralsetinib based on the results in lung and thyroid RET-altered cancers. [285].

It is essential to promote the research of targeted gene panels and the analysis of tumor tissue and/or circulating tumor cells (CTCs) and cell-free circulating tumor DNA (ctDNA) through liquid biopsies. Furthermore, reliable predictive biomarkers to foresee the response or resistance to certain therapies are becoming critical in order to choose the correct treatment for each patient. All these studies are available through NGS techniques and the difference lies in the quantity of genes analysed: approximately 20 to 500 genes in panels, 22,000 human protein-coding genes in whole-exome sequencing (WES) and 3.3 billion bases of the human genome in whole-genome sequencing (WGS) [281,286].

The improvement of technology has led to consider comprehensive genomic profiling (CGP) of tissue through NGS as the standard of care for sequencing multiple tumor samples. The clinical potential of NGS, with its short- and long-read applications, covers from diagnostic to prognosis by using small pre-surgical biopsies or even fine-needle-aspirations. It is not only important its utility in treatment, by identifying drug targets, but also the resistance alterations. These applications have been incorporated to different clinical studies at different stages: initial screening, disease progression or as longitudinal monitoring of molecular variations. This is possible since NGS may be used in tumor biopsies but also in ctDNA or CTCs, which helps to avoid monitoring the mutations with repeated biopsies and the subsequent risks and discomfort that this causes to patients [281,287].

Initially, TKIs were very unselective, addressing several different TKRs. This is known as multi-TKIs (MKI), which can range from targeting a small number of kinases to being highly promiscuous. It is thought that inhibiting different pathways at the same time would provide superior efficacy and safety profiles. Although this approach remains to be true, novel selective TKIs have been developed in the past years in order to avoid the off-target effects of non-selective MKIs, and, also, hoping that specifically targeting driver mutations can result in better outcomes and less toxicity when compared to non-selective MKIs. For instance, preliminary results from selective RET inhibitors have shown impressive results in lung cancer and MTC [288] However, the use of highly selective TKIs does not always lead to tumor response, and even when it does, a considerable number of patients relapse due to the appearance of acquired resistance mutations. This is why there is still a constant need of research in order to overcome this resistance so that patients can be offered subsequent treatment lines.

There is a physiologic rationale to combine immunotherapy with TKIs in cancer treatment. Angiogenesis inhibition seems to potentiate the antitumor activity of checkpoint inhibitors by increasing immune cells infiltration into tumors. In murine models, simultaneous inhibition of VEGF and PD-1 synergistically increased T-cell infiltration into tumor microenvironment. The initial efforts to combine TKIs and immunotherapy, which took place in metastatic RCC, yielded poor results due to unacceptable toxicity. The arrival of more selective VEGF inhibitors, such as axitinib, allowed the design of randomized clinical trials combining this molecule with checkpoint inhibitors, such as pembrolizumab and avelumab, showing a benefit in overall survival when compared to standard treatment, therefore becoming the treatment of choice in patients with metastatic RCC [260,261,289,290,291,292,293].

The combination of TKIs with chemotherapy has also been explored. Despite showing promising results in preclinical models, the combination of TKIs with chemotherapy (platinum-based) in EGFR-mutated non-small cell lung cancer, does not seem to be beneficial in terms of PFS and OS [294].

The combination of TKIs has also being evaluated in the past few years. Cell line models suggested that using specific TKIs could enhance intracellular concentrations of a targeted TKI, hence overcoming resistance and increasing efficacy. This rationale has been put to test in several clinical trials, especially in EGFR-mutated NSCLC, with the aim to overcoming or delaying drug resistance in these patients. In a recent study, third-generation EGFR inhibitor osimertinib—current first-line treatment in this setting—has been combined with first-generation gefitinib, showing a similar toxicity profile, with survival outcomes yet to be published. In the same subset of patients, the phase Ib TATTON trial tested the combination of osimertinib with MEK1/2 inhibitor selumetinib or MET inhibitor savolitinib, showing acceptable tolerability [295,296,297,298]. Cancer is a dynamic reality and tumor drivers to be targeted may change. Biologic heterogeneity found within the primary tumor and metastases is another challenge to face for tailor-made treatment.

## 6. Conclusions

TKRs play a key role in the molecular pathways that lead to cell mitosis and differentiation. Therefore, genetic alterations in TKRs provide the cell with a survival advantage, which leads to tumorigenesis. The inhibition of these targets using specific agents has proven to be successful in cancer treatment. In recent years, increasingly selective molecules have been developed showing very satisfactory survival outcomes in clinical trials. Moreover, combination regimes (with other TKIs or immunotherapy) seem to have a synergistic effect and to further ameliorate the survival of cancer patients. It is imperative to standardize tumor genotyping in order to offer the most selective and effective treatment against specific molecular targets.

## Figures and Tables

**Table 1:** Tyrosine kinase receptors, its ligands and most representative functions. Adapted from *Molecular biology of the cell,* by Alberts B et al. [1].

Ligand	Receptors and Representative Examples	Most Representative Functions	Cellular and Tissue Distribution
EGF (Epidermal Growth Factor)	EGFR (HER1), HER2, HER3, HER4	Stimulates survival, growth, proliferation, and differentiation of various cell lines	- Plasma membrane and cell junctions. - Wide expression: epithelial, endothelial, neuronal and glial, bone, adipose, liver, and cardiovascular cells.
Insulin	Insulin receptor	Regulates carbohydrate metabolism and protein synthesis	- Vesicles and plasma membrane. - All human cells, especially pancreas
IGF (Insulin-like Growth Factor)	IGF-1R	Stimulates cell growth and survival. Regulation of growth in young people and has anabolic effects in adults.	- Vesicles and plasma membrane. - Detected in all human cells.
NGF (Nerve Growth Factor)	NTRK1 (TrkA, TrkB, TrkC)	Stimulates neural growth and differentiation	- Plasma membrane, vesicles and cytosol. - Adrenal gland, blood, central and peripheral nervous systems.
PDGF (Platelet-Derived Growth Factor)	PDGFRα, PDGFRβ	It stimulates survival, growth, proliferation and migration of various cellular subtypes.	- Nucleoplasm, plasma membrane, cell junctions (α), vesicles and additionally in Golgi apparatus (β) - Wide expression, especially ovarian (α)
GM-CSF (Granulocyte Macrophage-colony stimulating factor)	GM-CSFR or GMRα/β	It stimulates the proliferation of monocytes and their differentiation to macrophages.	- Golgi apparatus and plasma membrane. - Wide expression, especially blood cells and placenta.
FGF (Fibroblast Growth Factor)	FGFR1, FGFR2, FGFR3, FGFR4	It stimulates the proliferation of various cell types and inhibits the differentiation of other types.	- Plasma membrane. - Detected in all human cells.
VEGF (Vascular Endotelial Growth Factor)	VEGFR-1 (FLT-1), VEGFR-2 (KDR), VEGFR-3 (FLT-4)	Stimulates angiogenesis.	- Plasma membrane, actin filaments (1), nucleoplasm, cell junctions (3) - Wide expression, especially blood compartment (mostly platelets), skeletal muscle and placenta
ALK (Anaplastic Lymphoma Kinase)	ALK (CD246)	Involved in the development and function of the central nervous system.	- Plasma membrane - Wide expression, especially the nervous system
GDNF (Glial cell-line derived neurotrophic factor)	GFRα1, GFRα2, GFRα3, GFRα4	Regulation of survival (dopaminergic neurons), growth of neurites, cell differentiation and migration.	- Plasma membrane Golgi apparatus and nucleoplasm (1), vesicles (2), cytosol (3) - Wide expression, especially brain, thyroid gland in GFRα2
SCF (Mast/Stem cell growth factor)	KIT (CD117)	It intervenes in processes such as the survival of melanocytes, hematopoiesis and gametogenesis.	- Plasma membrane - Hematopoietic stem cells, germ cells, melanocytes, and Cajal cells of the gastrointestinal tract, epithelial cells in skin adnexa, breast, and subsets of cerebellar neurons
Ephrin	Eph receptors	Guides cell and axon migration; angiogenesis.	- Plasma membrane, endoplasmic reticulum, cytosol - Wide expression, especially in the nervous system and injured tissues.
Gas6, Protein S.	TAM family (Tyro3, MerTK, Axl)	Cell growth, survival, differentiation. Regulation of systemic immunity	- Plasma membrane and additionally in vesicles and actin filaments - Wide expression

**Table 2:** EGFR and HER2 inhibitors, target and main clinical indications or trials.

Name (Code) Trade Name	Targets	Approved Clinical Indications or Clinical Trial Study
**Gefitinib** (ZD1389) Iressa	EGFR	NSCLC
**Erlotinib** (OSI-774) Tarceva	EGFR	NSCLC
**Afatinib** (BIBW2992) Tovok	Erb1/2/4	NSCLC
**Osimertinib** (AZD-9291) Tagrisso	EGFR	NSCLC
**Dacomitinib** (PF299804) Visimpro	Pan-HER	NSCLC
**Saracatinib** (AZD0530)	EGFR, Src	Phase II trial (NCT00752206, NCT00607594, NCT01267266) for osteosarcoma, gastric, prostate and other solid tumors
**Lapatinib** (GW572016) Tykerb	EGFR/HER2	Breast cancer
**Neratinib** (HKI-272) Nerlynx	HER2	Breast cancer
**Icotinib**	EGFR	NSCLC (China)
**Poziotinib** (HM781-36B)	EGFR/HER2/HER4	Phase II trial (NCT02979821) for NSCLC
**Tarloxotinib** (TH4000)	EGFR/HER2	Phase II trial (NCT03805841) for NSCLC and solid tumors harboring ERBB/NRG1 gene fusions
**Lazertinib** (YH25448)	EGFR	Phase III (NCT04248829) trial for NSCLC
AZD3759	EGFR	Phase II/III (NCT03653546) trial for NSCLC.
**Pyrotinib** (SHR-1258)	EGFR/HER2	Phase I trial (NCT02500199) for HER2 positive solid tumors.
**Avitinib** (AC0010MA)	EGFR	Phase I/II (NCT02330367) clinical trial for NSCLC
**Sapitinib** (AZD8931)	EGFR/HER2/HER3	Phase I/II trial (NCT01862003) for colorectal cancer.
**Rociletinib** (CO-1686)	EGFR	Phase III (NCT02322281) for NSCLC
TAS6417	EGFR/HER2/HER3	Phase I/IIa trial (NCT04036682) for NSCLC
**Varlitinib** (ASLAN001)	EGFR/HER2/HER4	Phase II/III (NCT03093870, NCT03130790) for billiard tract cancer, gastric cancer and hepatocarcinoma (Phase Ib; NCT03499626)
**Olmutinib** (HM61713)	EGFR	Phase Ib (NCT04510415) and phase II (NCT03228277) clinical trials for NSCLC
**Nazartinib** (EGF816)	EGFR	Phase I/II (NCT02108964) clinical trial for NSCLC
**Mavelertinib** (PF-06747775)	EGFR	Phase II trial (NCT02349633) for NSCLC
**Naquonitinib** (ASP8273)	EGFR	Phase I (NCT02113813) trial for NSCLC
**Ibrutinib** (PCI-32765) Imbruvica	EGFR, BTK	Phase I/II (NCT02321540) trial for NSCLC, MCL, CLL.
EAI001	EGFR	Preclinical
EAI045	EGFR	Preclinical

**Table 3:** ALK, ROS1 and NTRK inhibitors, target and main clinical indications or trials.

Name (Code) Trade Name	Targets	Approved Clinical Indications or Clinical Trial Study
**Crizotinib** (PF 2341066) Xalkori	ALK, MET, ROS1	NSCLC
**Ceritinib** (LDK378) Zykadia	ALK, IGF-1R, ROS1	NSCLC
**Alectinib** (CH5424802)Alecensa	ALK, RET	NSCLC
**Brigatinib** (AP 26113) Alunbrig	ALK, ROS1, EGFR	NSCLC
**Lorlatinib** (PF-06463922) Lorbrena	ALK, ROS1	NSCLC
**Ensartinib** (X-396)	ALK, MET, Axl, ABL, EPHA2 LTK, ROS1, SLK	Phase II (NCT01625234) and phase III (NCT02767804) trials for NSCLC
**Merestinib** (LY2801653)	NTRK, MET, ROS1, FLT3, Axl	Phase II trial (NCT02920996) for NSCLC and solid tumors with NTRK fusion proteins.
**Belizartinib** (TSR-011)	ALK, TRKA/B/C	Phase I/II trial for solid tumors and lymphomas with NTRK fusion proteins
**Entrectinib** (RXDX-101) Rozlytrek	TRKA/B/C, ROS1	Solid tumors with NTRK fusion proteins, ROS1-positive NSCLC
**Larotrectinib** (LOXO-101) Vitrakvi	TRKA/B/C	Solid tumors with NTRK fusion proteins
**Repotrectinib** (TPX-0005)	ROS1, TRKA/B/C, ALK	Phase I/II trial (NCT03093116) for solid tumors with NTRK fusion proteins and ROS1-positive NSCLC
**Taletrectinib** (DS-6051b)	ROS1, TRKA/B/C	Phase I trial (NCT02675491) for solid tumors with NTRK and ROS1 fusion proteins.
**Selitrectinib** (BAY2731954)	TRKA/B/C	Phase I/II trial (NCT03215511) for solid tumors with NTRK fusion proteins.
BMS-754807	TRKA/B, Insulin receptor, MET	Phase II trial (NCT01225172) for breast cancer.

**Table 4:** FGFR inhibitors, target and main clinical indications or trials.

Name (Code) Trade Name	Targets	Approved Clinical Indications or Clinical Trial Study
**Erdafitinib** (JNJ-42756493) Balversa	FGFR1/2/3/4	Urothelial bladder cancer
**Nintedanib** (BIBF-1120) Vargatef	FGFR1/2/3	Idiopathic pulmonary fibrosis, NSCLC
**Pemigatinib** (INCB054828) Pemazyre	FGFR1/2/3	Cholangiocarcinoma
**Rogaratinib** (BAY 1163877)	FGFR1/2/3	Under development in SQCLC (NCT03762122), breast cancer (NCT04483505), urothelial carcinoma (NCT03473756), sarcoma GIST (NCT04595747), and gastric cancer (NCT04077255)
**Vofatamab** (B-701)	FGFR 3	Under development in urothelial carcinoma (NCT03123055, NCT02401542)
**Infigratinib** (BGJ398)	FGFR1/2/3	Under development in urothelial carcinoma (NCT04197986), breast cancer (NCT04504331), cholangiocarcinoma (NCT03773302), and glioblastoma (NCT04424966)
**Derazantinib** (ARQ 087)	FGFR1/2/3	Under development in urothelial carcinoma (NCT04045613), gastric cancer (NCT04604132) and cholangiocarcinoma (NCT03230318)
AZD4547	FGFR1/2/3	Under development in NSCLC (NCT01824901), breast cancer (NCT01202591), gliomas (NCT02824133), urothelial carcinomas (NCT02546661) and gastro-esophageal cancer (NCT01457846)
Debio 1347	FGFR1/2/3	Under development in breast cancer (NCT03344536) and other solid tumors (NCT03834220)

**Table 5:** c-KIT and PDGFR inhibitors, target and main clinical indications or trials.

Name (Code) Trade Name	Targets	Approved Clinical Indications or Clinical Trial Study
**Imatinib** (STI-571) Glivec	PDGFR, c-Kit, v-Abl	GIST, CML (Chronic Myeloid Leukemia), ALL (Acute lymphocytic leukemia), dermatofibrosarcoma protuberans, myelodisplasic síndrome, leukemias.
**Avapritinib** (BLU-285) Ayvakit	PDGFR, c-Kit	GIST
**Ripretinib** (DCC-2618) Qinlock	c-Kit, PDGFR	GIST
**Amuvatinib** (MP-470)	c-Kit, PDGFR, Flt3	Under development in NSCLC (NCT01357395) and other solid tumors (NCT00894894)
**Dasatinib** (BMS-354825) Sprycel	c-Kit, Abl, Src	CML

**Table 6:** MET inhibitors, target and main clinical indications or trials.

Name (Code) Trade Name	Targets	Approved Clinical Indications or Clinical Trial Study
**Capmatinib** (INC280) Tabrecta	MET	NSCLC, under development in solid tumors harboring MET mutations.
**Tepotinib** (EMD 1214063)	MET	Under development in NSCLC (NCT02864992) and colorectal cancer (NCT04515394)
**Glumetinib** (SCC244)	MET	Under development in NSCLC (NCT04270591) and other solid tumors (NCT03457532)
**Savolitinib** (AZD6094)	MET	Under development in solid tumors harboring MET mutations.
AMG 337	MET	Under development in clear cell sarcoma (NCT03132155) and other solid tumors (NCT01253707)
**Bozitinib** (PLB-1001)	MET	Under development in gliomas (NCT02978261), NSCLC (NCT04258033), renal cell carcinoma and hepatocellular carcinoma (NCT03655613), and other solid tumors (NCT03175224)
**Merestinib** (LY2801653)	MET, AXL, DDR1, DDR2	Under development in NSCLC (NCT02920996), biliary tract cancer (NCT02711553) and other solid tumors (NCT03027284)
**Tivantinib** (ARQ197)	MET	Under development in multiple solid tumors harboring MET mutations.
**Foretinib** (GSK1363089)	MET, Tie-2, VEGFR3	Under development in several solid tumors.
**Crenolanib** (CP-868596)	PDGFR, FLT3	Under development in GIST (NCT02847429), glioma (NCT01393912), and esophagogastric carcinoma (NCT03193918)

**Table 7:** RET inhibitors, target and main clinical indications or trials.

Name (Code) Trade Name	Targets	Approved Clinical Indications or Clinical Trial Study
**Selpercatinib** (LOXO-292) Retevmo	RET	Thyroid cancer and NSCLC
**Pralsetinib** (BLU-667) Gavreto	RET	NSCLC
BOS172738	RET	Under development in solid RET-mutated tumors (NCT03780517)
TAS0953/HM06	RET	Under development in solid RET-mutated tumors
TPX-0046	RET, SRC	Under development in solid RET-mutated tumors (NCT04161391)

**Table 8:** Antiangiogenics, target and main clinical indications or trials.

Name (Code) Trade Name	Targets	Approved Clinical Indications or Clinical Trial Study
**Sunitinib** (SU011248) Sutent	VEGFR1/2/3, FLT3, Kit, PDFGRβ	GIST, RCC, pNET
**Pazopanib** (GW786034) Votrient	VEGFR1/2/3, PDGFRα/β, FGFR cKIT.	RCC, soft-tissue sarcoma.
**Tivozanib** (AV-951) Fotivda	VEGFR1/2/3	RCC
**Axitinib** (AG013736) Inlyta	VEGFR1/2/3, c-KIT	RCC
**Cabozantinib** (BMS-907351) Cabometyx, cometriq	c-MET, VEGFR2, RET, TRKA, TRKB and Axl.	RCC, HCC, MTC
**Lenvatinib** (E7080) Lenvima	VEGFR1/2/3, FGFR1/2/3/4, PDGFRα, c-KIT, RET	RCC, DTC, HCC
**Sorafenib** (BAY43-9006), Nexavar	VEGFR1/2/3, PDGFRβ, Flt-3, c-KIT, RET, Raf	HCC, DTC, RCC
**Regorafenib** (BAY73-4506) Stivarga	VEGFR1/2/3, Raf, TIE-2, PDGFR, RET, CSF-1	CRC, GIST, HCC
**Nintedanib** (BIBF1120) Vargatef	VEGFR1/2/3, PDGFRα/β), FGFR1/2/3, TRK, Flt-3 RET	NSCLC
**Vandetanib** (ZD6474) Caprelsa	VEGFR, EGFR and RET.	MTC
**Apatinib** (YN968D1)	VEGFR2	Gastric cancer (China)
**Fruquintinib** (HMPL-013)	VEGFR1/2/3	CRC (China)
**Anlotinib** (AL3818)	VEGFR2/3, c-Kit	NSCLC (China)
**Motesanib** (AMG-706)	VEGFR1/2/3, c-Kit, RET, PDGFR	Under development in several solid tumors
**Linifanib** (ABT-869)	VEGFR1/2, CSF-1R, FLT3, c-Kit	Under development in several solid tumors
**Glesatinib** (MGCD-265)	VEGFR1/2/3, MET, RON, Tie-2	Under development in NSCLC (NCT02544633) and other solid tumors
**Cediranib** (AZD2171)	VEGFR1/2/3, c-Kit, PDGFRβ, FGFR1	Under development in several solid tumors
**Dovitinib** (CHIR-258)	FGFR1/2/3, VEGF, c-Kit, FLT3	Multiple clinical trials mainly in renal cell carcinoma (phase III), breast cancer, hepatocellular cancer, endometrial cancer and GIST
**Tivozanib** (AV-951)	VEGFR1/2/3, EphB2, PDGFR	Under development in several solid tumors
**Vatalanib** (PTK787)	VEGFR1/2/3, PDGFR, c-Kit	Under development in several solid tumors
**Brivanib** (BMS-540215)	VEGFR1/2, FGFR1	Under development in several solid tumors
**Apatinib** (YN968D1)	VEGFR2, RET	Under development in several solid tumors
**Ponatinib** (AP24534)	VEGFR2, PDGFR, FGFR1	Under development in several solid tumors
**LY2874455**	VEGFR2, FGFR1/2/4	Under development in solid tumors (NCT01212107)
**Golvatinib** (E7050)	VEGFR2, MET	Under development in several solid tumors
**Telatinib** (BAY 57-9352)	VEGFR2/3, c-Kit, PDGFR	Under development in gastric cancer (NCT00952497, NCT03817411) and other solid tumors (NCT03175497)
**Lucitanib** (E-3810)	VEGFR1/2/3, FGFR1/2	Under development in several solid tumors
**Sulfatinib**	VEGFR1/2/3, FGFR1, CSF1R	Under development in neuroendocrine tumors (NCT04579679), thyroid cancer (NCT04524884), biliary tract carcinoma (NCT03873532) and other solid tumors (NCT04579757, NCT02549937)

**Table 9:** Other TKIs, target and main clinical indications or trials.

Name (Code) Trade Name	Targets	Approved Clinical Indications or Clinical Trial Study
**Bosutinib** (SKI-606)	SRC, STAT3	Under development in breast cancer (NCT03854903) and other solid tumors (NCT03023319)
**Pexmetinib** (ARRY-614)	MAPK, Tie-2	Under development in solid tumors (NCT04074967)
**Dubermatinib** (TP-0903)	Axl	Under development in solid tumors (NCT02729298)
**Bemcentinib** (BGB324)	Axl	Under development in breast cancer (NCT03184558), pancreatic cancer (NCT03649321), and SNCLC (NCT03184571)
**Sitravatinib** (MGCD516)	Axl, VEGFR3	Under development in several solid tumors
**Ningetinib** (C31H29FN4O5)	Axl, c-MET, VEGFR2	Under development in NSCLC (NCT03758287)

## Abbreviations

MDPI	Multidisciplinary Digital Publishing Institute
DOAJ	Directory of open access journals
TLA	Three letter acronym
LD	linear dichroism

## Appendix A

**Table A1:** Main TKR mutations distributed by various types of solid and hematological tumors. [124,126,151,152,156,171,179,195,198,199,237,238,299,300,301,302].

TKR Mutations	Most Representative Tumors	Other Described Alterations in Tumors
EGFR (primary mut.) *Exon 19 (del), exon 21 (L858R, L861Q), exon 18 (G719X), exon 20 (ins)* EGFR (secondary mut.) *Exon 20 (T790M), D761Y, T854A, L747S*	NSCLC (15–20%) NSCLC	Colorectal carcinoma (EGFR S464L, G465R, I491M, EGFR S492R) Pancreatic carcinoma Head and neck cancer (EGFR amplifications) Glioma (EGFR A289V, R108K, G598V, T263P)
HER2 mut. *Exon 20 (ins)*	NSCLC (1–3%)	Breast cancer (HER2 amplification negative; HER2 D769Y, D769H, R896C, V777L, G309E, V842I, S310F, S310Y, C311R; HER2 inframe deletion (755-759), inframe insertion (780GSP), inframe insertion (781GSP)
ALK rearrangement *EML4-ALK, KIF5B-ALK, KLC1-ALK, HIP1-ALK* ALK (secondary mut.) *Exon 23 (L1196M, S1206Y), exon 25 (G1269A), exon 22 (I1171T), V1180L, F1174L*	NSCLC (5–6%) NSCLC (5–6%)	Anaplastic large cell lymphoma (other not ALK L1196M; ALK F856S, A348D) Glioblastoma (ALK fusions) Inflammatory myofibroblastic tumors (ALK C1156Y) Diffuse large B-cell lymphoma (ALK G1269A) Esophageal squamous cell carcinoma (ALK L1152R) Colorectal carcinoma (ALK F1245C)
FGFR1-4	NSCLC (Squamous) (FGFR1 amplif., 17%) SCLC (FGFR1 amplif., 6%) Breast cancer (FGFR1 amplif., 15%) Cholangiocarcinoma (FGFR2 fusions) Urothelial carcinoma (FGFR2-3 fusions) Gastric cancer (FGFR2 amplif., 5–10%)	Cholangiocarcinoma (other not FGFR2 fusion; FGFR2 N549H, V564F, K659M, L617V, K641R, R565A) Bladder cancer (oncogenic mutations others not FGFR3 fusions) Endometrial carcinoma (FGFR2 M536I, M538I, I548V, N550, E566G, L618M, K660E, S252W, N550K, V565I) NSCLC (Squamous) (other not FGFR1 amplification; FGFR2 W290C, S320C, K660; FGFR3 S249C, G691R) Myeloma multiple (FGFR3 K650, Y373C, V555M)
c-KIT mut. *Exon 11, exon 9, exon 17, exon 13*	GIST (80–85%)	AML (KIT D816V, N822K) Systemic mastocytosis (KIT D816V) Thymic carcinoma (KIT H697Y) Cutaneous melanoma (KIT amplif.)
PDGFR mut. *Exon 18, exon 12, exon 14*	GIST (6–8%)	Cutaneous melanoma (PDGFRA V658A, P577S, R841K, H845Y, G853D) Renal carcinoma (PDGFRA overexpression) Myelodisplasic syndrome (PDGFRA fusion) Dermatofibrosarcoma protuberans (translocation t(17:22))
MET skipping mut. *Exon 14*	NSCLC (3%)	Renal carcinoma (MET H1112R) NSCLC (MET amplif.) Colorectal, gastric carcinoma (MET amplif.)
ROS1	NSCLC, cholangiocarcinoma, gastric carcinoma (ROS1 fusions, 1–2%)	NSCLC (ROS1 G2032R) Inflammatory myofibroblastic tumor
RET	Papillary thyroid carcinoma (RET rearrangement, 5–40%)	MEN-2 syndrome, sporadic medullary thyroid carcinoma (RET mutations)

## Appendix B

**Table A2:** Inhibitory concentrations (IC50) in nmol for targets with multi-targeted tyrosine kinase inhibitors [303,304,305,306,307,308,309,310,311,312,313].

	VEGFR1	VEGFR2	VEGFR3	PDFGRa	PDGFRb	c-KIT	RET	Flt-3	MET	CSF-1R	FGFR1	FGFR2	FGFR2	FGFR4	Axl
Sunitinib	2	9	17	7	8	7	10	250	-	890	830	-	-	-	9
Pazopanib	10	30	47	71	81	74	-	-	-	-	140	-	130	80	-
Tivozanib	30	6	15	40	49	78	-	2550	550	-	525	-	1250	1400	-
Axitinib	0.1	0.2	0.1–0.3	5	1.6	1.7	>1000	>1000	-	73	100	-	-	-	-
Cabozantinib	-	0.035	-	-	234	4.6	5.2	11.3	1.3	-	5294	-	-	-	7
Lenvatinib	22	4	5.2	25	39	0.7	12	-	1900	-	46	-	-	43	-
Sorafenib	26	90	20	-	57	68	-	33	-	-	580	-	-	-	-
Regorafenib	13	4.2	46	-	22	7	1.5	-	-	-	202	-	-	-	-
Nintedanib	34	21	13	59	65	-	-	26	-	-	69	37	100	610	-
Vandetanib	-	40	110	-	-	-	100	-	-	-	-	-	-	-	-
